# BTN3A1 promotes tumor progression and radiation resistance in esophageal squamous cell carcinoma by regulating ULK1-mediated autophagy

**DOI:** 10.1038/s41419-022-05429-w

**Published:** 2022-11-22

**Authors:** Wenjing Yang, Bo Cheng, Pengxiang Chen, Xiaozheng Sun, Zhihua Wen, Yufeng Cheng

**Affiliations:** 1 Department of Radiation Oncology, Qilu Hospital of Shandong University, Cheeloo College of Medicine, Shandong University, Jinan, People’s Republic of China; 2 Laboratory of Basic Medical Sciences, Qilu Hospital of Shandong University, Cheeloo College of Medicine, Shandong University, Jinan, People’s Republic of China; 3 Department of Radiation Oncology, Shandong Cancer Hospital and Institute, Cheeloo College of Medicine, Shandong University, 250012 Jinan, Shandong P. R. China

**Keywords:** Radiotherapy, Tumour biomarkers, Mitophagy

## Abstract

Radiotherapy is one of the most effective treatments for esophageal squamous cell carcinoma (ESCC); however, radioresistance is a clinical problem that must urgently be solved. Here, we found that butyrophilin subfamily 3 member A1 (BTN3A1) is upregulated in ESCC tumor tissues compared with nontumor tissues. We also evaluated BTN3A1 expression in patients with ESCC receiving adjuvant radiotherapy. The results demonstrated that BTN3A1 upregulation predicts a poor prognosis for ESCC patients. BTN3A1 overexpression promotes ESCC cell proliferation in vitro and in vivo. Moreover, BTN3A1 knockdown sensitized ESCC cells to radiation. We further explored the mode of death involved in BTN3A1-mediated radioresistance. Previous studies have shown that apoptosis, autophagy, necrosis, pyroptosis and ferroptosis are important for the survival of ESCC cells. We performed an RT-PCR array and western blotting (WB) to identify the mode of death and revealed for the first time that BTN3A1 promotes cell radioresistance by activating autophagy. In addition, by performing immunoprecipitation and mass spectrometry analyses, we found that BTN3A1 regulated the expression of UNC-51-like autophagy activating kinase 1(ULK1) and promoted its phosphorylation to subsequently initiate autophagy. Chromatin immunoprecipitation (ChIP) and luciferase reporter assay results indicated that BTN3A1 is a novel direct target of hypoxia inducible factor-1α (HIF-1α). HIF-1α, a transcription factor, promotes BTN3A1 transcription upon irradiation. Overall, the present study is the first to show that BTN3A1 plays a key role in radioresistance and that targeting BTN3A1 might be a promising strategy to improve radiotherapy efficacy in patients with ESCC.

## Introduction

Esophageal cancer is a common malignant tumor of the digestive tract. Notably, 572,000 new cases of esophageal cancer and 509,000 deaths occur worldwide every year [1]. Esophageal squamous cell carcinoma (ESCC) is one of the main subtypes, accounting for approximately 90% of esophageal cancers in China [2]. The 5-year overall survival (OS) rate of patients with ESCC is only 15% to 25% [3]. According to NCCN guidelines, radiotherapy has become the preferred treatment for locally advanced esophageal cancer. However, many factors affect the efficacy of radiotherapy. Among them, radiotherapy resistance seriously influences the prognosis of patients with esophageal cancer. Only 15.6–16.0% of patients with EC undergoing radiotherapy achieve a clinical complete response [4]. An understanding of the molecular mechanism of radioresistance is crucial. Gene mutations have been shown to affect the responses of cancer cells to radiation [5]. We propose that the difference in the expression of some key genes in patients with esophageal cancer may contribute to radiotherapy resistance, and effective targeted therapies are urgently needed to help clinicians tailor treatment plans for patients with ESCC.

Human butyrophilin subfamily 3 member A (BTN3A), also known as CD277, includes isoforms BTN3A1, BTN3A2, and BTN3A3 and is structurally associated with B7 costimulatory molecules [6]. Each BTN3A isoform consists of an extracellular N-terminal Ig variable (V) domain and a near-membrane Ig constant (C) region connected to a single transmembrane domain [6, 7]. The extracellular regions of the three subtypes are structurally similar, sharing 95% homology [8]. In recent years, BTN3A family members have emerged as important molecules modulating the function of Vγ9Vδ2 T cells [9, 10]. In particular, the B30.2 intracellular domain plays an important role in this process [11]. However, only the intracellular B30.2 domain of BTN3A1 directly binds phosphorylated antigens (pAgs) through a positively charged pocket to activate Vγ9Vδ2 T cells [12]. A humanized anti-BTN3A antibody has been evaluated in an interventional, nonrandomized phase I/II clinical study in patients with relapsed/refractory advanced-stage disease [13]. In addition, BTN3A1 is expressed in many tumors, such as ovarian cancer, bladder cancer, breast cancer, renal cell carcinoma and pancreatic ductal adenocarcinoma [14–17]. However, its role in these tumors is unclear, and the prognostic role of BTN3A1 in different cancers varies substantially.

In our research, we revealed that BTN3A1 is upregulated in ESCC tissues compared with adjacent tissues. High BTN3A1 expression predicted a poor prognosis and was related to recurrence after postoperative radiotherapy (PORT) for locally advanced ESCC. We further explored the function of BTN3A1. BTN3A1 promoted ESCC cell proliferation and enhanced radioresistance in vitro and in vivo. BTN3A1 depletion impeded DNA replication, cell colony formation and motility. Moreover, upon exposure to radiotherapy, BTN3A1 knockdown significantly enhanced DNA damage and reduced cell proliferation. To date, the mode of BTN3A1-mediated tumor cell death has not been studied. We revealed for the first time that BTN3A1 promotes tumor cell radioresistance by activating autophagy. We explored the relationship between BTN3A1 expression and radiation-mediated autophagy in vitro and in vivo. Subsequently, we used a public database and mass spectrometry to reveal that BTN3A1 could bind to UNC-51-like autophagy activating kinase 1 (ULK1) and regulated the expression and phosphorylation of ULK1. We also investigated the mechanism of BTN3A1 overexpression in ESCC upon irradiation. The chromatin immunoprecipitation (ChIP) and luciferase reporter assay results indicated that BTN3A1 is a novel direct target of HIF-1α. Our study might provide a basis for the development of alternative strategies, such as combination radiotherapy and BTN3A1 inhibitor therapy, for patients with BTN3A1-high ESCC.

## Results

### BTN3A1 upregulation is associated with a poor prognosis in ESCC patients

**Fig. 1:**
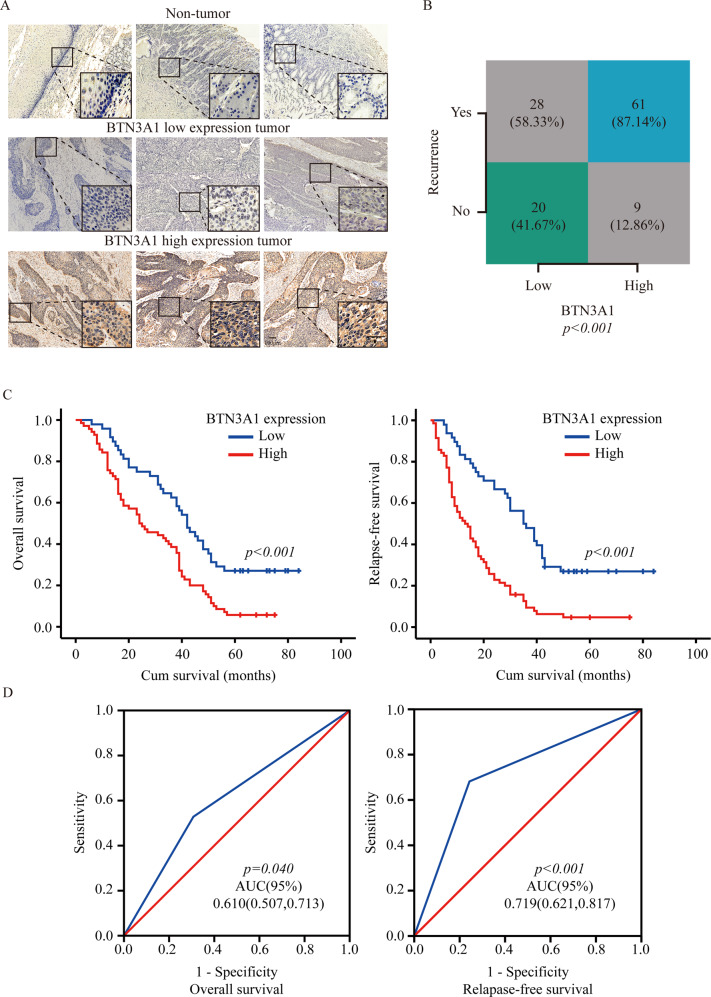
BTN3A1 upregulation is associated with a poor prognosis in ESCC. **A** IHC Images of BTN3A1 staining in biopsy specimens from ESCC patients. Nuclei were counterstained with haematoxylin. Scale bar: 100 μm, 50 μm. **B** The relationship of BTN3A1 expression in the tumor and recurrence rate in ESCC patients samples, Chi-square test. **C** Kaplan–Meier curves for the OS and RFS of patients with high BTN3A1 expression (*n* = 70) and low BTN3A1 expression (*n* = 48). The *p* value was calculated using the log-rank test. **D** ROC curve analysis of OS and RFS. AUC values were estimated to assess the predictive ability of BTN3A1 expression.

We collected samples from 118 patients and examined BTN3A1 expression in tumor and adjacent nontumor tissues by performing immunohistochemical (IHC) staining. As shown in Fig. 1A, BTN3A1 was mainly located in the cytoplasm and only a few were distributed on the cell surface, and its expression was significantly increased in cancer tissues. Based on the average levels of BTN3A1 expression, samples were separated into 2 groups (high BTN3A1: *n* = 70; low BTN3A1: *n* = 48). The clinicopathological variables for all patients are presented in Supplementary Table S1. Lymph node metastasis (N stage) (*p* = 0.001) and tumor invasion (T stage) (*p* = 0.032) were significantly correlated with high BTN3A1 expression (Table 1). Furthermore, among the 118 patients, 51 experienced in-field recurrence after PORT (43.2%). High BTN3A1 expression was positively correlated with the rates of in-field recurrence and distant metastasis (Fig. 1B, *p* < 0.001). Additionally, Kaplan–Meier curve analysis showed that lower BTN3A1 expression was related to longer OS and relapse-free survival (RFS) (Fig. 1C). Univariate and multivariate analyses confirmed that BTN3A1 was an independent prognostic indicator for RFS (*p* < 0.05) and OS (*p* < 0.05; Table 2). Receiver operating characteristic (ROC) curves were analyzed for the Cox regression model. The area under the curve (AUC) value of BTN3A1 for OS was 0.610 (*p* = 0.04) and for RFS was 0.719 (*p* < 0.001; Fig. 1D). Moreover, using a tissue microarray, we investigated the correlation between BTN3A1 expression and the clinicopathological features of patients with ESCC who were not treated with radiotherapy. The BTN3A1 protein level was evaluated in ESCC tumor and nontumor tissues (Fig. S1A). A significant correlation was observed between BTN3A1 upregulation and T stage (*p* = 0.002), N stage (*p* < 0.001), and TNM stage (*p* < 0.001; Supplementary Table S2). The Kaplan–Meier curve and survival analysis showed that BTN3A1 upregulation predicted a poor prognosis (Fig. S1B and Supplementary Table S3).

**Table 1:** The correlation of clinicopathological characteristics of ESCC patients receiving radiotherapy with BTN3A1 expression.

Clinicopathological features	BTN3A1 expression		*P* value
	Low (*n* = 48)	High (*n* = 70)	
Age			0.359
<65	36	47	–
≥65	12	23	–
Gender			0.085
Female	13	10	–
Male	35	60	–
Differentiation			0.697
Well	4	6	–
Moderate	27	34	–
Poor	17	30	–
Location			
Cervical	2	0	0.292
Up	4	4	–
Middle	31	45	–
Lower	11	21	–
T stage			**0.032**
T1-2	23	20	–
T3-4	25	50	–
N stage			**0.001**
N0	33	26	–
N1-3	15	44	–
AJCC stage			0.069
I	6	2	–
II	23	30	–
III	19	38	–
Recurrence state			**0.002**
In-field recurrence	15	36	–
Metastasis	13	25	–
Others	20	9	–

*P* value calculated by the *χ*^2^ test or Fisher’s exact test; Values in bold signify *p* < 0.05, which is considered significant.

**Table 2:** Univariate and Multivariate analyses of prognostic factors for OS and RFS in patients receiving radiotherapy with ESCC.

		OS				RFS		
Variable	Univariate analysis	Multivariate analysis			Univariate analysis	Multivariate analysis		
	*P* value	*P* value	HR	95% CI	*P* value	*P* value	HR	95% CI
Age	0.329	–	–	–	0.150	–	–	–
Gender	0.114	–	–	–	0.056	–	–	–
Location	0.757	–	–	–	0.911	–	–	–
T stage	**0.002**	0.576	1.220	0.607−2.450	**0.001**	0.968	0.986	0.495−1.965
N stage	**<0.001**	0.880	1.067	0.460−2.475	**<0.001**	0.432	0.678	0.257−1.786
Differentiation	0.074	–	–	–	0.174	–	–	–
AJCC stage	**<0.001**	0.678	0.752	0.196−2.889	**<0.001**	0.741	0.799	0.211−3.030
Recurrence state	0.514	–	–	–	0.313	–	–	–
BTN3A1 expression (low vs high)	**<0.001**	**0.016**	2.181	1.157−4.113	**<0.001**	**0.001**	3.140	1.594−6.184

Values in bold signify *p* < 0.05, which is considered significant.

*CI* confidence interval, *ESCC* esophageal squamous cell carcinoma, *RFS* relapse-free survival, *HR* hazard ratio, *OS* overall survival, *T* tumor, *N* lymph node metastasis.

### BTN3A1, a proto-oncogene, promotes ESCC survival in vitro and in vivo

**Fig. 2:**
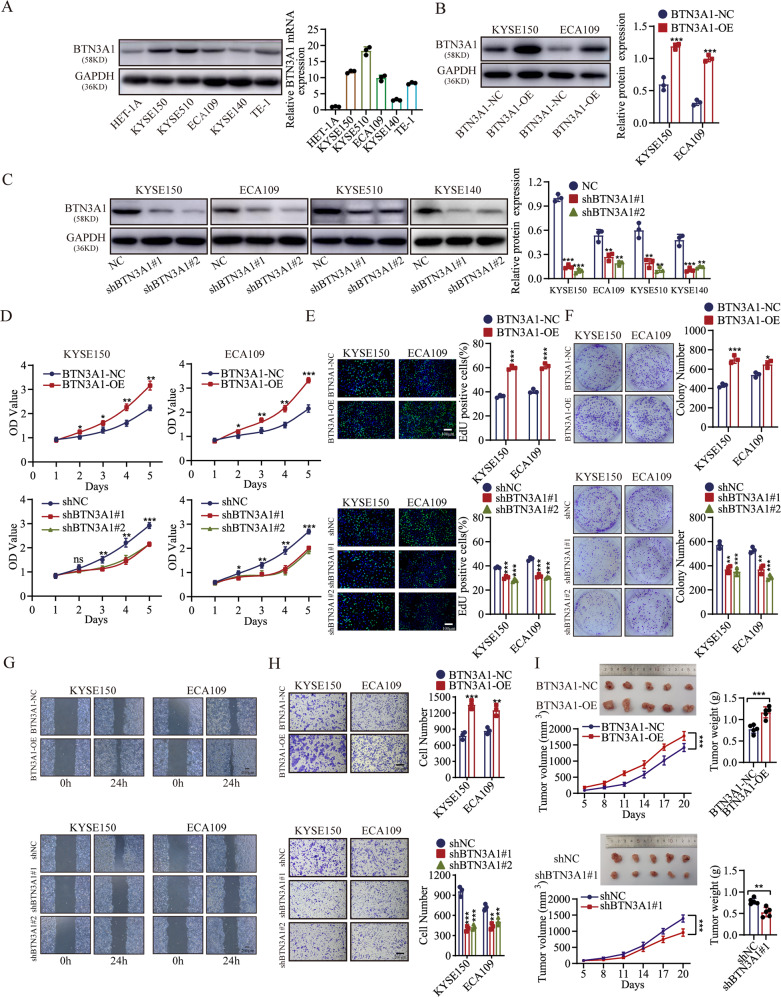
BTN3A1 promotes ESCC cell growth in vitro and in vivo. **A** The protein and mRNA expression of BTN3A1 in a normal esophageal epithelial cell (HET-1A) and human ESCC cells (KYSE150, KYSE510, ECA109, KYSE140, and TE-1). GAPDH was used as an internal control. **B**, **C** Effects of BTN3A1 overexpression and knockdown were confirmed by WB. **D** CCK-8 assays were performed to determine the effects of BTN3A1 overexpression and knockdown on the proliferation of KYSE150 and ECA109 cells. **E** Effect of BTN3A1 on DNA synthesis in KYSE150 and ECA109 cells. The cells were fluorescently stained with EdU (green). The nucleus was stained with Hoechst 33342 (blue). Scale bar: 100 µm. Quantification of the EdU-positive cells is shown in the right panel. **F** Effect of BTN3A1 overexpression and knockdown on colony formation. The cells were seeded into six-well plates at a density of 1000 cells per well, and cultured for 10 d, and then stained with crystal violet. **G** Wound healing assays of BTN3A1-overexpression and –knockdown cells. Scale bar:200 µm. **H** Transwell assays of BTN3A1-overexpression and - knockdown cells. Representative pictures are shown on the left, and quantifications on the right in **H**. Scale bar:200 µm. **I** Transplanted xenografts derived from KYSE150 cells transfected with BTN3A1-NC and BTN3A1-OE (top panel) and shBTN3A1#1 and shNC (bottom panel) constructs were established in BALB/c nude mice (*n* = 5). Tumor volumes and weights were measured (top panel, images of the removed xenografts; bottom-left panel, tumor size; bottom -right panel, weight). All data are presented as the means ± SD from three independent experiments; and *p < 0.05, **p < 0.01, ***p < 0.001 Student’s *t* test.

Research on BTN3A1 has mainly focused on its binding to pAgs and presentation to Vγ9Vδ2 T cells [18, 19], but there is no relevant report on its role in ESCC. In this study, BTN3A1 was detected in ESCC cell lines (KYSE150, KYSE510, ECA109, KYSE140, and TE-1) and a normal esophageal squamous cell line (HET-1A) using WB and RT‒PCR (Fig. 2A). Moreover, we examined the localization of BTN3A1 in cells. Immunofluorescence (IF) staining showed that BTN3A1 was mainly located in the cytoplasm of ESCC cells, and only a few were distributed on the cell surface. (Fig. S2A). KYSE150 and ECA109 cells with BTN3A1 upregulation or downregulation were established (Fig. 2B, C). BTN3A1 overexpression facilitated cell proliferation, while its downregulation impeded cell proliferation, as shown in Cell Counting Kit-8 (CCK-8) assays (Fig. 2D). 5-Ethynyl-2′-deoxyuridine (EdU) staining showed a higher DNA replication rate in BTN3A1-overexpressing cells (Fig. 2E). Subsequently, we conducted colony formation assays, and the results indicated that BTN3A1-overexpression accelerated cells colony formation (Fig. 2F). Furthermore, wound healing assays and transwell migration assays were performed to evaluate the cell motility (Fig. 2G, H). BTN3A1 overexpression enhanced the malignancy of tumor cells. The same results were confirmed in animal models. BTN3A1 promoted xenograft tumor growth, as reflected by an increase in tumor mass and heavier tumor weight (Figs. 2I and S2B).

### BTN3A1 knockdown sensitizes ESCC cells to radiation in vitro and in vivo

**Fig. 3:**
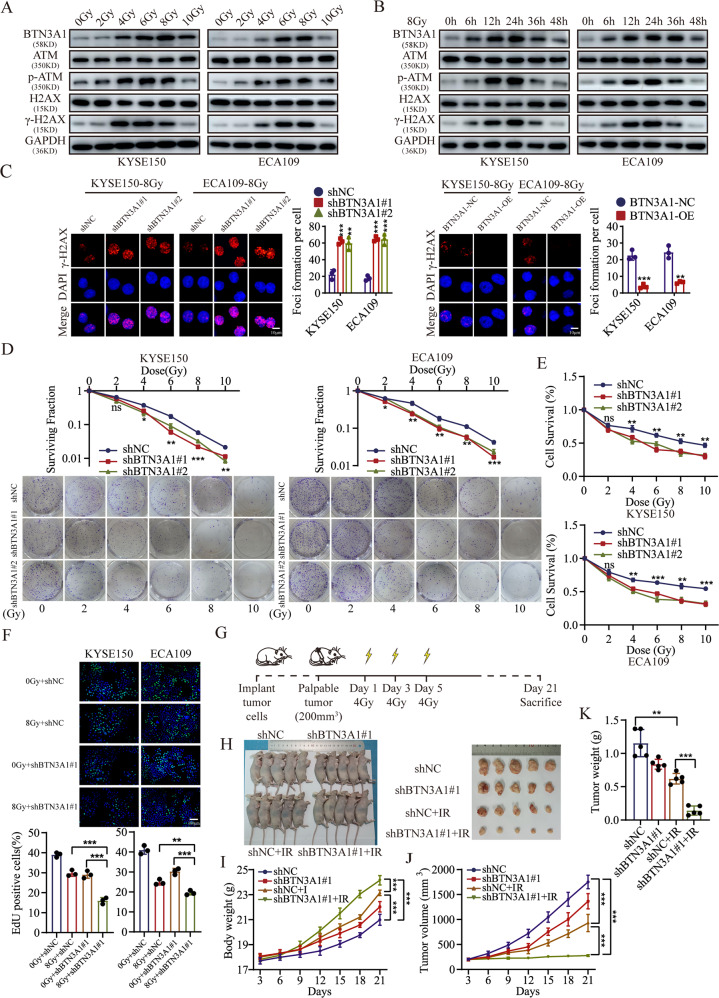
BTN3A1 knockdown inhibits radioresistance in vitro and in vivo. **A** WB showing of BTN3A1, ATM, H2AX, p-ATM, and γ-H2AX protein level after exposure to the different radiation doses (0, 2, 4, 6, 8, or 10 Gy) and subsequent culture for 24 h. **B** Level of BTN3A1, ATM, H2AX, p-ATM and γ-H2AX were detected in KYSE150 and ECA109 cells after radiation exposure (8 Gy) and culture for different times (0–48 h). **C** IF was used to examine γ-H2A.X foci in ESCC cells that were treated with radiation (8 Gy) and cultured for 24 h. **D**, **E** Colony formation assays and CCK8 assays were performed to evaluate cell colony formation ability and the survival of BTN3A1-knockdown cells after radiation exposure (0, 2, 4, 6, 8, or 10 Gy). These data are representative of three independent experiments and are presented as means ± SD (*n* = 3); Student’s *t* tests. **F** Effect of BTN3A1 knockdown on DNA synthesis. KYSE150 and ECA109 cells treated with or without radiation (8 Gy) were fluorescently stained with EdU (green). The nucleus was stained with Hoechst 33342 (blue). The percentage of EdU-positive cells was calculated and is shown in the bottom panel. Scale bar: 100 µm. **G** Schematic of the in vivo experiment. KYSE150 cells with stable knockdown of BTN3A1 were injected subcutaneously. Once tumors reached a volume of ~200 mm^3^, the mice were exposed to 4 Gy of radiation on days 1, 3, and 5. On day 21, the mice were euthanized. **H** Representative images of xenografts from the indicated treatment groups. **I** The body weight of nude mice was measured at every 3 days. Means± SD (*n* = 5). **J** Tumor growth curves were compared using ANOVA. **K** Tumor weight was measured at the end of the experiment. Means± SD (*n* = 5); Student’s *t* tests; ns, no significance, *p < 0.05, **p < 0.01, ***p < 0.001.

We further determined the relationship between BTN3A1 expression and radiation. Radiation exerted positive effects on the level of BTN3A1 protein and increased the expressions of p-ATM and γ-H2AX (Fig. 3A). Additionally, we detected the time-dependent response of BTN3A1 expression and found that the increase of BTN3A1 was in an irradiation time-dependent manner. BTN3A1 expression began to increase at 6 h after irradiation (Fig. 3B). The statistical analysis of the BTN3A1, p-ATM, and γ-H2AX protein levels is shown (Fig. S3A, B). We further detected γ-H2AX levels in BTN3A1-KD and BTN3A1-OE cells after IR. WB assays indicated that BTN3A1 knockdown predisposed ESCC cells to radiation-induced DNA damage in a time-point dependent manner (Fig. S3C), whereas BTN3A1 overexpression made ESCC cells insensitive to radiation-induced DNA damage (Fig. S3D). Meanwhile, IF staining showed that the number of γ-H2AX foci was significantly increased in BTN3A1-KD cells (Fig. 3C). The colony formation assay showed that the clonogenic potential of KYSE150 and ECA109 cells infected with the lentivirus carrying the shRNA targeting the BTN3A1 gene was significantly reduced upon irradiation (Fig. 3D). The detailed radiobiological parameters, which confirmed the results, are shown in Supplementary Table S4. Consistent with these results, the CCK-8 assay showed that the survival rate of BTN3A1-knockdown cells was less than that of the control ESCC cells (Fig. 3E). EdU staining also revealed that BTN3A1 knockdown resulted in the radiosensitization of ESCC cells (Figs. 3F and S4A). Next, a BTN3A1 plasmid (shMT, a synonymous mutation) was constructed and transfected into KYSE150 cells to rescue BTN3A1 expression after knockdown. BTN3A1 expression was restored in knockdown cells (Fig. S4B), and radiation resistance was also restored (Fig. S4C–F and Supplementary Table S5).

We generated a xenograft mouse model using KYSE150 cell lines with stable BTN3A1 knockdown, and the specific tumor area was irradiated to validate whether BTN3A1 knockdown sensitizes ESCC cells to radiation in vivo (Fig. 3G). Radiotherapy moderately suppressed control group tumor growth compared with non-radiotherapy treatment (*p* < 0.01) while prominently inhibiting the growth of BTN3A1-knockdown tumors (*p* < 0.001; Fig. 3H). The body weight of mice subjected to both treatments increased rapidly (Fig. 3I). Tumors grew slower with both ionizing radiotherapy (IR) treatment and BTN3A1 depletion (*p* < 0.001) (Figs. 3J, K and S4G). Based on the above results, we suggest that BTN3A1 knockdown sensitizes ESCC cells to IR and that BTN3A1 inhibitor could be developed as drugs to improve radiotherapy efficacy in ESCC patients.

### BTN3A1 knockdown decreases the level of autophagy in ESCC cells

**Fig. 4:**
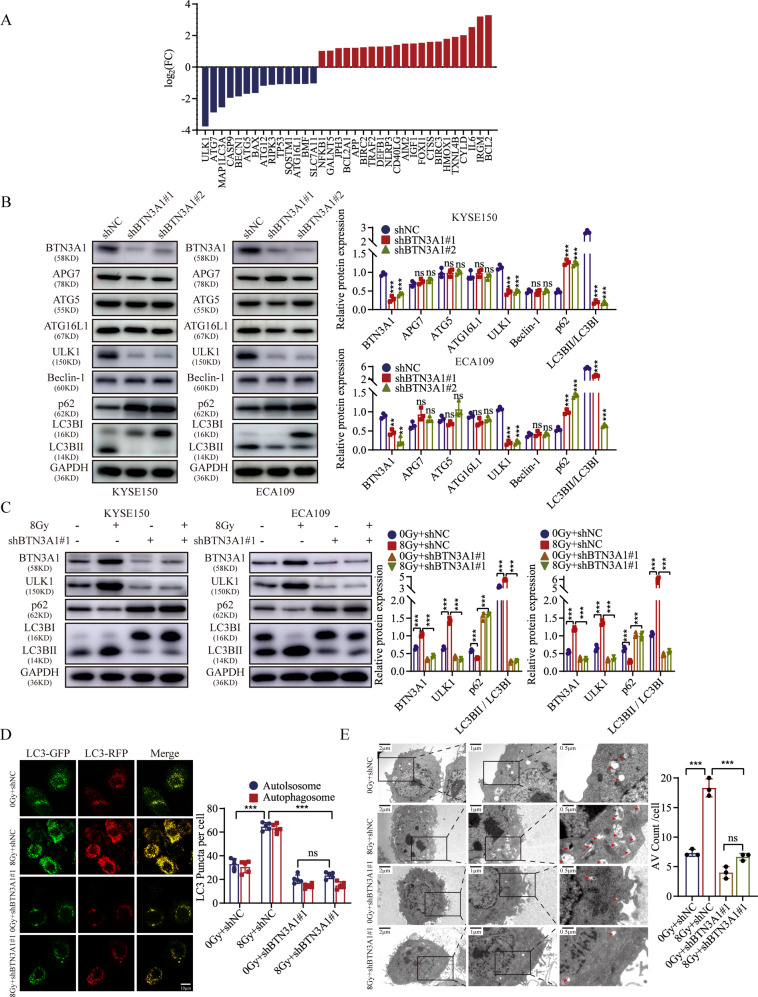
BTN3A1 knockdown suppresses autophagy in ESCC cells. **A** 35 differentially expressed genes were identified in KYSE150 cells transfected with shBTN3A1 or shNC by death-related qPCR array. **B** Immunoblots of the key autophagy-related proteins in cells with BTN3A1 knockdown. **C** Control and BTN3A1-knockdown cells were treated with IR (8 Gy) and cultured for 24 h, followed by immunoblotting with the indicated antibodies. These data are representative of three independent experiments. ***p < 0.001 compared to the control group. **D** Control and BTN3A1-KD KYSE150 cells were co-transfected with mRFP-GFP-LC3B then treated with or without radiation (8 Gy). The confocal microscopy analysis is shown. Scale bar: 10 µm. **E** Autophagosomes were observed by transmission electron microscopy. Red arrows indicated autophagosomes formation. Scale bar: 2 µm. The data are presented as the means ± SD from three independent experiments. ns, no significance, *p < 0.05, **p < 0.01 and ***p < 0.001.

We used an RT‒PCR array to identify the mode of death involved in the effects of BTN3A1. When the shBTN3A1 group was compared with the control group, 35 significantly differentially expressed genes were identified, and these genes are shown in a histogram (Fig. 4A). Among all the genes related to specific modes of cell death, ULK1 was identified as one of the most relevant genes, which was shown in the volcano plot in Fig. S5A. Furthermore, the protein expression of the autophagy-, apoptosis-, and pyroptosis-related genes was also measured using WB (Figs. 4B, and S5B, C). We found that cells with BTN3A1 knockdown exhibited decreased ULK1 and microtubule-associated protein 1 light chain 3 beta (MAP1LC3B/LC3B) expression compared with control cells. We next explored the potential role of BTN3A1 in radiation-mediated autophagy. The expression of ULK1 and LC3BII /LC3BI was decreased, and the expression of sequestosome 1 (p62/SQSTM1) was increased in BTN3A1 knockdown cells compared with cells carrying a control shRNA after irradiation (8 Gy; Figs. 4C and S5D). We explored the effects of BTN3A1 on autophagosome (yellow puncta) and autolysosom (red puncta) formation by transfecting KYSE150 cells with tandem mRFP-GFP-LC3 lentivirus vectors. The data showed that reduced BTN3A1 expression reversed the autophagy flux that was activated by radiotherapy in ESCC cells (Figs. 4D and S5E). Transmission electron microscopy (TEM) was used to observe the ultrastructure of KYSE150 cells. Autophagic vacuoles (AVs) were counted in 12 randomly selected cells from each section. BTN3A1 depletion significantly decreased the number of AVs in irradiated cells compared with vector control cells (*p* < 0.001, Figs. 4E and S5F). Bafilomycin A1 (BafA1), which is a known inhibitor of the latter stages of autophagy that blocks degradation of p62, was used to determine whether BTN3A1 regulates autophagosome formation or autophagic flux. BTN3A1 increased the conversion of LC3B-I to LC3B-II and the accumulation of mRFP-GFP-LC3 puncta in KYSE150 cells. However, BafA1 inhibited the function of BTN3A1 by preventing the degradation of LC3BII and autophagic flux (Fig. S5G, H). Collectively, these results indicate that BTN3A1 participates in the process of autophagy, and that its knockdown reduced the level of radiation-induced autophagy in ESCC cells.

### BTN3A1 promotes cell radioresistance by activating autophagy

**Fig. 5:**
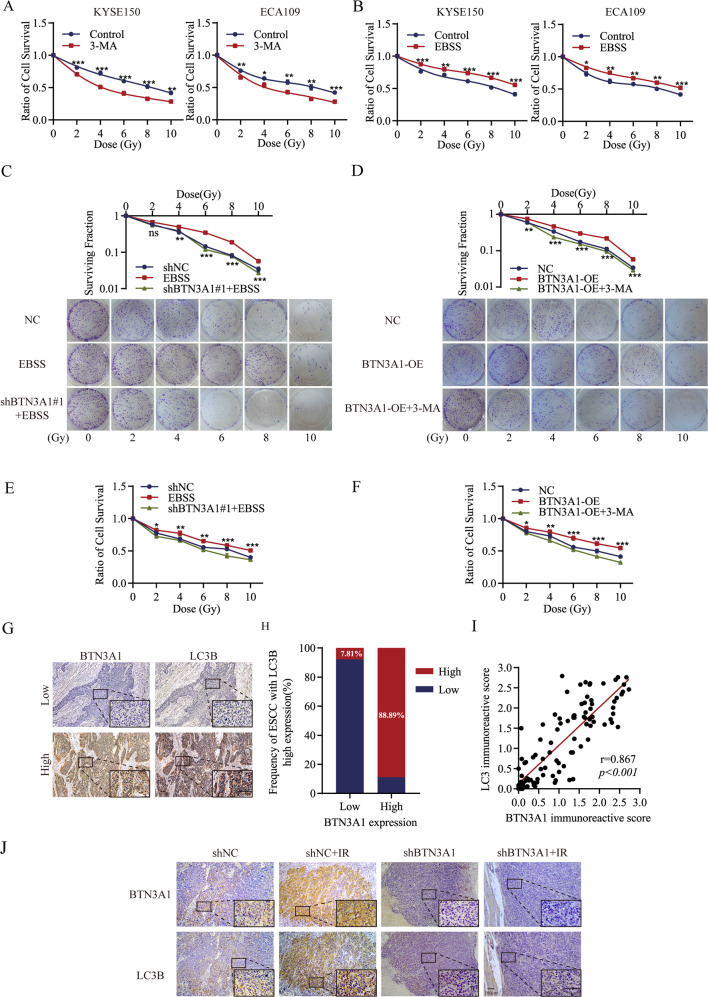
BTN3A1 knockdown sensitizes ESCC cells to irradiation by inhibiting autophagy. **A**, **B** CCK-8 assays were performed to measure the relative viability of KYSE150 and ECA109 cells treated with 3-MA or EBSS after exposure to the different radiation doses (0–10 Gy). **C**, **D** Colony formation assays were performed to evaluate cell colony formation ability after radiation exposure (0, 2, 4, 6, 8, and 10 Gy) and culture cells for 10 days under EBSS (**C**) or 3-MA (**D**), respectively. **E**, **F** CCK-8 assays were performed to evaluate cell survival after radiation exposure and culture cells for 24 h under EBSS (**E**) or 3-MA (**F**), respectively. These data are representative of three independent experiments. Means±SD (*n* = 3); t Student’s t tests; **p < 0.01,***p < 0.001. **G**–**I** Paraffin ESCC sections were used for IHC staining of the BTN3A1 and LC3B protein. Scale bar: 100μm. Correlation between the expression of the BTN3A1 and LC3B proteins. r, Pearson’s correlation coefficient. **J** Representative images of IHC staining for BTN3A1 and LC3B in xenograft tumors tissues. Scale bar, 100 µm, *p < 0.05, **p < 0.01 and ***p < 0.001.

Based on accumulating evidence, autophagy contributes to the radiation resistance of cancer cells [20]. In our study, we revealed that treatment with 3-methyladenine [21] (3-MA, an autophagy inhibitor)enhanced ESCC cell sensitivity to radiation(Fig. 5A). We induced starvation (using Earle’s balanced salt solution, EBSS) to increase the level of autophagy and found that radiation resistance was significantly increased in ESCC cell (Fig. 5B). Colony formation were performed to address whether BTN3A1 modulated radiation resistance in ESCC via the autophagy pathway. KYSE150 cells treated with EBSS were less sensitive to radiation than control cells. Intriguingly, when cells treated with EBSS were transfected lentivirus with a BTN3A1-specific shRNA, the sensitivity was increased to some extent and was not different from that of the control group (Fig. 5C). KYSE150 cells transfected with BTN3A1 plasmids were more resistant to IR than control cells, and the cells treated with 3-MA, were resensitized to IR (Fig. 5D). The detailed radiobiological parameters of the cells are shown in Supplementary Table S6. Furthermore, the results were confirmed by CCK-8 assays (Fig. 5E, F). Taken together, our results indicate that shBTN3A1-induced radiosensitization is at least partially due to a decreased level of autophagy.

Subsequently, we investigated the relationship between BTN3A1 expression and autophagy in ESCC samples. IHC staining revealed that LC3B protein expression was positively correlated with BTN3A1 expression in tumor tissues (*r* = 0.867, *p* < 0.001; Fig. 5G–I). Additionally, radiation increased LC3B staining, but this change was attenuated with BTN3A1 knockdown in transplanted tumors (Fig. 5J).

### BTN3A1 triggers autophagy mediated by ULK1

**Fig. 6:**
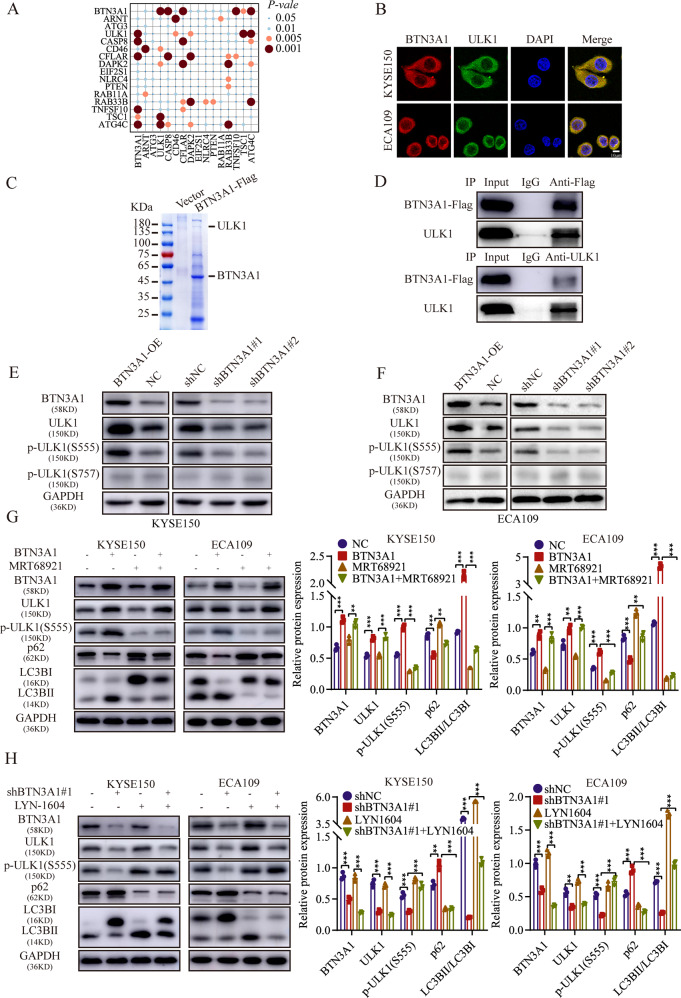
BTN3A1 regulates autophagy through the ULK1 pathway. **A** A bubble chart using the HADb Human Autophagy database revealed that BTN3A1 expression was significantly correlated with ULK1 expression. ESCC samples were obtained from TCGA database. *p* < 0.001. **B** IF staining showing the localization of BTN3A1 (red) and ULK1 (green) in KYSE150 and ECA109 cells. The nuclei were stained with DAPI (blue). Scale bar, 10 µm. **C** The empty vector or BTN3A1-Flag plasmid was transfected into KYSE150 cells and cultured for 24 h. Cell lysates was measured by immunoprecipitation with anti-FLAG beads, and the proteins interacting with BTN3A1 were analyzed using mass spectrometry. An image of Coomasie brilliant blue staining is shown. **D** Co-immunoprecipitation of BTN3A1 and ULK1 was performed in KYSE150 cells. **E**, **F** KYSE150 and ECA109 cells were harvested 48 h after transfection with the BTN3A1-specific plasmid (BTN-OE), BTN3A1 shRNA lentivirus (shBTN3A1#1/#2), or corresponding empty vectors. The lysates were immunoblotted and quantified using ImageJ software. **G** The protein level of autophagy markers (ULK1, p-ULK1, p62, and LC3B) were examined using WB. Autophagy induced by BTN3A1 overexpression was inhibited by treatment with 1 μM MRT68921in the ESCC cell lines. **H** Autophagy suppressed by BTN3A1 knockdown was rescued by treatment with LYN-1604. These data are representative of three independent experiments. Student’s *t* tests; * *p* < 0.05, ** *p* < 0.01, and ***p < 0.001.

To investigate the mechanism by which BTN3A1 participates in autophagy, gene expression profile data from The Cancer Genome Atlas (TCGA) database were analyzed. BTN3A1 expression was closely related to the autophagy-related genes ULK1, caspase8 (CASP8), FADD-like apoptosis regulator (CFLAR), TNF superfamily member 10 (TNFSF10) and autophagy-related 4 C cysteine peptidase (ATG4C; *p* < 0.001; Fig. 6A). Meanwhile, we also selected independent ESCC case cohorts from the Gene Expression Omnibus (GEO) database (i.e., 50 ESCC cases, accession numbers GSE161533, GSE20347 [22], and GSE17351 [23]; Fig. S6A). Combining TCGA database and GEO database analysis results, we speculated that BTN3A1 expression was related to ULK1. IF staining revealed the co-localization of BTN3A1 and ULK1 in ESCC cells (Fig. 6B). Subsequently, we performed co-immunoprecipitation (Co-IP) in KYSE150 cells, and the proteins pulled down by specific antibody were identified using mass spectrometry analysis. ULK1 was detected in only the BTN3A1 group but not in the vector group (Fig. 6C and Supplementary Table S7). The interaction between BTN3A1 and ULK1 was also confirmed by Co-IP in KYSE150 cell (Fig. 6D) and ECA109 cell (Fig. S6B). BTN3A1-mediated autophagy may be related to the ULK1 gene. According to some reports, ULK1 is phosphorylated by multiple upstream kinases, such as mTOR and AMPK, on different sites and affects its kinase activity. In our study, BTN3A1 overexpression regulated the expression of ULK1 and promoted its phosphorylation at Ser555 rather than Ser757. Moreover, BTN3A1 knockout suppressed the phosphorylation of ULK1 at Ser555 in KYSE150 (Figs. 6E and S6C) and ECA109 cells (Figs. 6F and S6D). WB was performed to determine the effect of BTN3A1 on the ULK1 pathway. Treatment with 1 μM MRT68921 inhibited ULK1 activity and reduced autophagy which stimulated by BTN3A1 overexpression. The results suggested that BTN3A1 induced autophagy through increasing ULK1 activity (Fig. 6G). In addition, the inhibition of autophagy by BTN3A1 knockdown was rescued by treatment with a ULK1 agonist (LYN-1604; Figs. 6H and S6E).

### Radiation increases BTN3A1 expression through a process mediated by HIF-1α

**Fig. 7:**
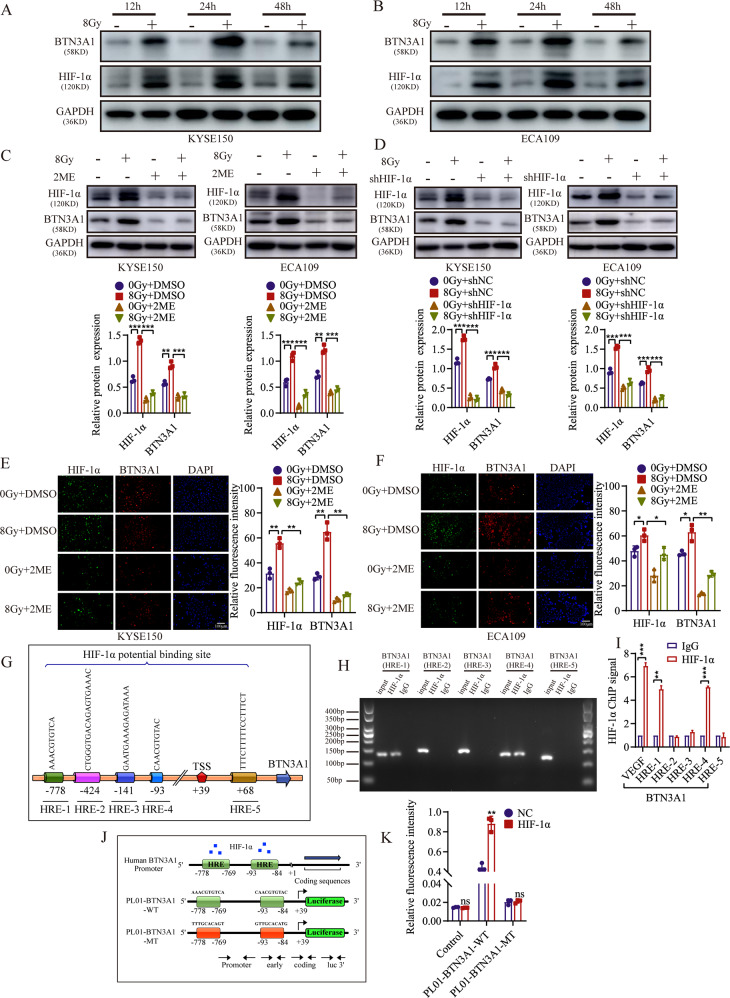
Radiation increases BTN3A1 expression through a process mediated by HIF-1α. **A**, **B** KYSE150 and ECA109 cells were treated with or without IR (8 Gy). Then cells were collected at different time points and immunoblotted with antibodies against BTN3A1, HIF-1α and GAPDH. **C** Cells were treated with 2.5 μM 2-ME (HIF-1α inhibitor), irradiated with 8 Gy X-ray, and collected 24 h later. Lysates were immunoblotted with antibodies against BTN3A1, HIF-1α and GAPDH. **D** Cells were transfected with HIF-1α shRNA lentivirus and irradiated with 8 Gy X-ray. After 24 h, cells were lysed and immunoblotted with antibodies against BTN3A1 and HIF-1α. **E**, **F** KYSE150 and ECA109 cells were exposed to 8 Gy radiation. After 24 h, the expression of BTN3A1 and HIF-1α was examined using IF staining. Scale bar = 100 μm. **G** Schematic of the BTN3A1 promoter. The predicted HIF-1α binding sites were marked as HRE-1, 2, 3, 4, and 5. **H** Binding of HIF-1α to the BTN3A1 promoter region in vitro was assessed using ChIP with anti- HIF-1α or anti-IgG antibodies in KYSE150 cells. **I** Input DNA purified by ChIP were measured using qRT-PCR. **J**, **K** Luciferase reporter assays using the BTN3A1 promoter in KYSE150 cells. Cells were co-transfected with a series of BTN3A1 mutant promoter plasmids and HIF-1α overexpression plasmids or the control plasmids. Luciferase activities were measured 48 h later. The data are presented as the means ± SDs; ns, no significant difference; *p < 0.05; **p < 0.01; ***p < 0.001.

Following radiotherapy, tumor oxygenation tends to increase. HIF-1α may be the source of signal transduction and a major determinant of tumor radiosensitivity [24]. Radiation exposure induces HIF-1α protein expression in pancreatic cancer and lung cancer [25, 26]. In our study, the preliminary results showed radiation increased BTN3A1 and HIF-1α expression (Fig. 7A, B). Statistical analysis of the BTN3A1 and HIF-1α protein levels is shown in Fig. S6F, G. Subsequently, the HIF-1α inhibitor 2.5 μM 2-methoxyestradiol (2-ME) was added to the cells. Radiation-induced BTN3A1 overexpression was suppressed (Fig. 7C). HIF-1α shRNA was transfected into KYSE150 and ECA109 cells to further confirm that HIF-1α is involved in irradiation-mediated BTN3A1 expression (Fig. 7D). The results revealed that IR may activate BTN3A1 in a HIF-1α-dependent manner. IF staining indicated that treatment with 2-ME resulted in significantly lower levels of BTN3A1 and HIF-1α than the control treatment (Fig. 7E, F). We also assessed the HIF-1α response in BTN3A1 knockdown cells. BTN3A1 expression did not affect the response of HIF-1α (Fig. S6H). BTN3A1 may be a downstream gene of HIF-1α.

**Fig. 8:**
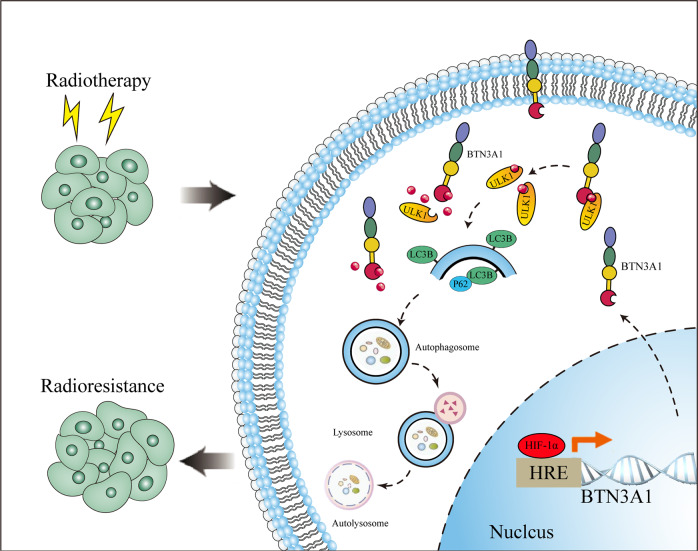
Working model of BTN3A1 the mechanism by which promotes radioresistance by modulating autophagy in ESCC cells. Radiation increased BTN3A1 expression through a pathway mediated by HIF-1α. BTN3A1 regulated the expression of ULK1 and promoted its phosphorylation to subsequently initiate autophagy.

To investigate whether BTN3A1 is a direct HIF-1α target gene, we first conducted chromatin immunoprecipitation-quantitative PCR (ChIP‒qPCR) assays to verify the five potential binding sites for HIF-1α in the BTN3A1 promoter predicted by Jaspar and hTF target (Fig. 7G, H). The ChIP assay showed a substantial increase in the binding of HIF-1α to the chromatin region of the BTN3A1(HRE-1) and BTN3A1(HRE-4) promoters (Fig. 7I). To further determine whether HIF-1α interacts with the BTN3A1 genomic sequences, KYSE150 cells were co-transfected with pM14 empty vector and pM14 HIF-1α vector, pL01 BTN3A1-hRluc/SV40, or pL01 BTN3A1-hRluc/SV40 MUT vectors (Fig. 7J). As shown in Fig. 7K, cells transfected with the HIF-1α vectors displayed more than twofold higher luciferase activity of the BTN3A1 reporter than cells not transfected with HIF-1α vectors. Based on these results, we conclude that BTN3A1 is a direct target gene of HIF-1α and that radiation might upregulate BTN3A1 expression by increasing HIF-1α levels. The proposed mechanism is summarized in Fig. 8.

## Discussion

Radiotherapy is the preferred treatment regimen for patients with locally advanced ESCC. However, radiation resistance is a pressing challenge. The human BTN3A1 protein has been linked to pAg-dependent activation of Vγ9Vδ2 T cells [19]. The activation of γδT cells may not be the only function of BTN3A1. A 2020 study reported that BTN3A1 binds to CD45N-mannosylated residues on αβT cells and blocks their antigen-specific activation, thus preventing adequate TCR activation [9]. The phosphorylation of PI3K, AKT, and ERK was affected by a monoclonal antibody 20.1 against BTN3A (-CD277) [7]. BTN3A1 also binds periplakin and RhoB GTPase in the cytoplasm and functions as a hub in the protein-protein interaction network associated with activation processes [27]. In addition, BTN3A1 is dysregulated in different types of human cancer, including breast cancer, ovarian cancer, bladder cancer, renal cell carcinoma, and pancreatic ductal adenocarcinoma [14–17].

In our study, we showed for the first time that BTN3A1 is highly expressed in ESCC tissues. High BTN3A1 expression was associated with lymph node metastasis and advanced T stage. Survival analyses suggested that BTN3A1 upregulation was an independent factor predicting a poor prognosis for ESCC patients. Moreover, BTN3A1 overexpression is correlated with poorer overall survival in ESCC patients receiving adjuvant radiotherapy. In addition, we found that elevated BTN3A1 expression promoted tumor cell growth in vitro and in vivo. To the best of our knowledge, the effect of BTN3A1 expression on radiotherapy resistance has not been reported previously. Here, radiation induced an increase in BTN3A1 expression, accompanied by DNA damage. BTN3A1 knockdown cells were more sensitive to IR than BTN3A1 wild-type cells based on the results from CCK-8 and colony formation assays. Next, we used a synonymous mutant plasmid to restore the expression of BTN3A1 in knockdown cells. The results showed that radiation resistance also increased with the recovery of BTN3A1 expression. Consistently, BTN3A1 knockdown reduced the radiosensitivity of ESCC cells in the animal model.

An understanding of the molecular mechanism underlying radioresistance will help improve the efficacy of radiotherapy. IR causes cell death by triggering cellular stress responses, including the generation of DNA damage responses, reactive oxygen species, endoplasmic reticulum stress, and mitochondrial damage [28]. However, radiation resistance may occur in two ways in cancer cells: through the activation of damage-repair signaling pathways [29, 30], or through the inhibition of death-related signaling pathways [31]. Previous studies have shown that apoptosis, autophagy, necrosis, pyroptosis and ferroptosis are important for the survival of ESCC cells [32–34]. We used PCR array assays and publicly available data to study the death mode involved in BTN3A1-mediated radioresistance. We knocked down BTN3A1 gene and examined the expression levels of apoptosis-, pyroptosis- and autophagy-related proteins in KYSE150 and ECA109 cells using WB. We found that BTN3A1 expression is closely related to autophagy.

Subsequently, we explored the potential role of BTN3A1 in radiation-mediated autophagy. Autophagy is a highly conserved catabolic process for maintaining energy homeostasis, in which cellular constituents such as damaged organelles and dysfunctional proteins are sequestered by autophagosomes and delivered to autolysosomes to be degraded and recycled [35, 36]. Our research revealed that the expression of the autophagy-related proteins ULK1 and LC3BII /LC3BI was decreased, and the expression of p62 was increased in BTN3A1 knockdown cells exposed to irradiation. Moreover, using laser confocal and transmission electron microscopy, we found that BTN3A1-KD reversed the autophagy flux activated by radiotherapy. BafA1, a known autophagy inhibitor, prevents the degradation of p62 and LC3BII and reduces LC3BII to LC3BI, thereby interrupting autophagic flux. We used BafA1 in the experiments to determine whether BTN3A1 regulates autophagosome formation or autophagic flux. Overexpression of BTN3A1 significantly induced mRFP-GFP-LC3 puncta accumulation, and lipid conjugation of free LC3-I to the autophagic membrane-associated LC3-II was increased in the extracts of cells overexpressing BTN3A1. After adding BafA1, autophagic flux was blocked, and the autophagic flux mediated by BTN3A1 was also significantly weakened. CCK-8 assays and colony formation assays also confirmed that BTN3A1 knockdown inhibited radiation-induced autophagy, and autophagy was identified as the core mechanism of radiosensitization.

In the initial stage of autophagy, the ULK1 protein kinase complex regulates the formation of autophagosomes [37]. Previous reports have confirmed that phosphorylation of ULK1 is important for autophagy induction [38, 39]. AMPK promotes autophagy by directly activating ULK1 through phosphorylation at Ser555. High mTOR activity prevents ULK1 activation by phosphorylating ULK1 at Ser757 and disrupting the interaction between ULK1 and AMPK. In our studies, we verified the co-localization of BTN3A1 with ULK1 by IF staining using confocal microscopy, and revealed for the first time that BTN3A1 could interact with ULK1 by Co-IP and mass spectrometry analyses. The WB results suggested that BTN3A1 regulates ULK1 expression and promotes its phosphorylation at Ser555. We also examined the effect of BTN3A1 on the ULK1 pathway. Treatment with MRT68921 reduced autophagy stimulated by BTN3A1 overexpression, suggesting that BTN3A1 induces autophagy by increasing ULK1 activity in ESCC cells. In addition, the inhibition of autophagy by BTN3A1 knockdown was rescued by treatment with a ULK1 agonist.

We also explored the mechanism of radiation-induced BTN3A1 ectopic expression. In a xenograft mouse model, BTN3A1 expression was significantly increased in tumors treated with radiation compared with tumors not treated with radiation. We speculated that IR alters the expression of BTN3A1. HIF-1α, a major transcription factor, has been reported to be associated with radiation-mediated hypoxia and is a major determinant of tumor radiosensitivity [40]. One study [41] showed that radiotherapy upregulated HIF-1α through an increase in the availability of oxygen and an improvement in oxidative stress in EC. HIF-1α increases vascular endothelial growth factor (VEGF) expression, which protects vascular endothelial cells from the cytotoxic effects of radiation. In our study, when BTN3A1 expression was elevated by radiotherapy, HIF-1α expression was also increased. We inhibited HIF-1α expression with inhibitors or lentivirus shRNA constructs and found that radiation-induced BTN3A1 overexpression was also suppressed. We also assessed the HIF-1α response upon IR in BTN3A1 knockdown cells. BTN3A1 expression did not affect the response of HIF-1α to radiation. These results confirmed that BTN3A1 may be a direct target gene of HIF-1α. Finally, ChIP and luciferase assays validated the sequences of the BTN3A1 promoter to which HIF-1α bound. We conclude that radiation can upregulate BTN3A1 expression by increasing HIF-1α levels.

Several limitations of the current study should be discussed. First, since few patients with locally advanced EC chose preoperative radiotherapy, strong conclusions could not be drawn from the analysis of PORT specimens. As a method to address this limitation, future multicenter studies using operative specimens with large sample sizes should be conducted to determine the BTN3A1 expression pattern in ESCC. Second, we must determine whether ULK1 is a conserved substrate of BTN3A1 and explore whether there are other phosphorylation sites involved.

Collectively, our present study reveals a BTN3A1-dependent mechanism that BTN3A1 overexpression promote cell growth and resistance to radiation. BTN3A1 knockdown sensitizes ESCC cells to radiation in vitro and in vivo. This is the first study to show that BTN3A1 confers radioresistance to ESCC cells by promoting autophagy. We also revealed that BTN3A1 is a novel factor that regulates radiation-induced autophagy by promoting ULK1 Ser555 phosphorylation. Furthermore, irradiation also upregulates BTN3A1 expression by increasing HIF-1α levels. Our findings provide a novel therapeutic target that can be used in combination with radiotherapy in ESCC patients, and this also suggests a possible scenario in which BTN3A1 upregulation could be a biomarker for selecting appropriate treatment regimens for ESCC patients.

## Material and methods

### Patients and specimens

A total of 118 ESCC and 30 noncancerous esophageal specimens, all of which were formalin-fixed paraffin-embedded (FFPE) samples from patients with detailed long-term follow-up clinical data, were obtained between 1 January 2011, and 30 December 2014, at Qilu Hospital of Shandong University. All patients had undergone radical esophagectomy with lymphadenectomy followed by radiotherapy for 5 weeks (DT: 50 Gy/25 fractions, 2 Gy/fraction, and 5 fractions/week). Patients with ESCC presenting with anastomotic leakage, systemic infection or other tumors were excluded. No patients had received preoperative chemo/radiotherapy. Staging of the disease was confirmed by independent pathologists based on the American Joint Committee on Cancer 8th edition. Patients without distant metastasis were selected for research. This study was approved by the Medical Ethics Committee of Qilu Hospital, Shandong University, and was performed in accordance with the Declaration of Helsinki of the World Medical Association. Informed consent was obtained from all patients. RFS was assessed from the day of the end of PORT to the date of in-field recurrence or distant metastasis. In-field was defined as within the 95% isodose line. Tumor recurrence was monitored by imaging examination systems or biopsy. A tissue microarray (Catalog ESC77, Superbiotek Pharmaceutical Technology Co.) was also used in this study.

### Cell culture and irradiation treatment

Human ESCC cells KYSE150 and ECA109 cells and a normal esophageal squamous cell line (HET-1A) were acquired from the China Center for Type Culture Collection (CCTCC, Wuhan, China). KYSE140, and KYSE510 were donated by Professor Yan Li from Sun Yat-Sen University Cancer Center. The cell lines were identified by the Genetic Testing Biotechnology Corporation (Suzhou, China). HET-1A cells were cultured in DMEM (Gibco, Life Technologies Inc., Grand Island, NY, USA), and the other cells were cultured in RPMI 1640 media (Gibco). The media were supplemented with 10% FBS (Gibco) and 1% antibiotics were added to the medium. All cells were maintained at 37 °C in a humidified chamber with 5% CO2. KYSE-150 cells and ECA109 cells (1×10^6^) were plated in 25cm^2^ culture flasks. When the cells reached ~70% confluence, the medium was replaced, and the cells were irradiated with 2 Gy of X-rays using a high linear accelerator (Varian 600 C, USA) at a dose rate of 100 cGy/min, with a 1.5-mm tissue compensation membrane. Following irradiation, cells were returned to the incubator. Irradiation (2 Gy) was repeated on days 2 and 3, and the cells were cultured for 4 days to recover. These procedures were repeated to achieve a total doses of 2 Gy, 4 Gy, 6 Gy, 8 Gy, and 10 Gy, respectively. When 90% confluence, the cells were trypsinized and subcultured into new flasks. Parental cells serving as the control (0 Gy) were trypsinized, counted and passaged under the same conditions without irradiation.

### Lentivirus infection and cell transfection

Lentiviral vectors carrying a human BTN3A1-specific shRNA (target sequence 1: 5′- GAGAGAGACATTCAGCCTATA-3′.Target sequence 2: 5′-CUAUUUGUCCAGCGUGAAA-3′) or a scrambled non-targeting control (sequence: 5′-TTCTCCGAACGTGTCACGT-3′) were purchased from Genechem (Shanghai, China). Vector information for GV112: is listed in related manuscript file. Lentiviral vectors carrying HIF-1α-specific shRNA (target sequence: 5′-CCAGCAGACTCAAATACAATT-3′) and a scrambled non-targeting control (sequence: 5′-ACAGAAGCGATTGTTGATC-3′) were purchased from GeneCopooeia (Shanghai, China). Vector information for CS-SH3295-LVRU6GP is listed in related manuscript file. The plasmids of pcDNA3.1-control and pcDNA3.1-BTN3A1 (sequence: 5′-CTTGGTACCGAGCTCGGATCC-3′) and pcDNA3.1-BTN3A1 (shMT) (sequence: 5′-TGAAAGGCACTCTGCTTACAATGAATGGAAAAAGGCCCTCTTCA-3′) plasmids were designed and synthesized by GeneMeditech (Shanghai, China). Vector information for pCDNA3.1 (+)-3×Flag-C is listed in related manuscript file. The plasmids were transfected into ESCC cells using Lipofectamine 3000 (Invitrogen, Carlsbad, CA, USA) and selected with neomycin. The stable clones were selected with puromycin. The efficiency of gene knockdown and overexpression was measured using RT-PCR.

### RNA extraction and quantitative RT‒PCR array

Total RNA was extracted using nuclear acid purification reagent (Fastagen, Shanghai, China) according to the manufacturer’s protocol. Approximately 2.0 μg of total RNA were used for cDNA synthesis using the High Capacity cDNA Reverse Transcription Kit (Thermo Fisher Scientific, USA) according to the manufacturer’s protocol. Relative gene mRNA expression was evaluated with quantitative RT-PCR using a QuantiFast SYBR Green PCR Kit (Thermo Fisher Scientific, USA). The sequences of the primers used are listed in Supplementary Table S8. RT-PCR arrays were designed to analyze Human Reactive Cell Death genes in ESCC cell line (Wcgene Biotechnology Corporation, China). GAPDH or β-actin was used as an internal standard for normalization. The reaction was completed using Light Cycler 480 (Roche). At least three biological replicates were examined.

### Cell proliferation assays

Cell proliferation was detected using the CCK-8 kit (Bioss, Beijing, China). Transfected ESCC cells (3 × 10^3^/well) were seeded into 96-well plates (Corning Incorporated, Corning, NY, USA) and exposed to a total of 2, 4, 6, 8, or 10 Gy with a 6-MV X-ray beam. After cultured for 24 h, 10 μl of the CCK-8 solution were added to each well containing 100 μl of serum-free medium. The cells were incubated for 2 h at 37 °C. The absorbance was read at 450 nm with a spectrophotometer (Tecan, Männedorf, Switzerland). The following formula was used to assess cell proliferation: (OD_experiment_−OD_blank_)/ (OD_control_−OD_blank_) × 100%. All assays were repeated three times.

### Colony formation assays

Cell survival was measured using a clonogenic assay. Cells with BTN3A1 knockdown or overexpression were plated into 6-well plates at a density of 1000 cells/well. After 10 days of incubation, cells were fixed with 4% paraformaldehyde and stained with 0.5% crystal violet. Visible colonies (more than 50 cells) were counted. The radiosensitivity was also determined using this assay. ESCC cells were seeded into six-well plates at a density of 1000, 1200, 1400, 1600, 1800, or 2000 cells/well and exposed to a total of 0, 2, 4, 6, 8, or 10 Gy (2 Gy per fraction), respectively. After 10 days of incubation, the colonies were stained and counted. Cell survival curves were obtained using Graph Pad Prism 8.0 software. Cell survival fraction (SF) was substituted into the following single-hit multitarget model: SF = 1–(1–e^ ^[−kD]^)^^N^. Radiobiological parameters (D0, Dq, N, and SF2) were calculated.

### EdU incorporation assay

Cells were plated into 24-well plates at a density of 1 × 10^5^cells/well and incubated for 24 h. The assay was performed using the Cell Light™ EdU Imaging Detection Kit (Ruibo Biotechnology, Guangzhou, China) according to the manufacturer’s instructions.

### Immunofluorescence

Cells were fixed with 4% paraformaldehyde for 15 min at room temperature and then were permeabilized with 0.5% Triton-X100 for 20 min. After blocking with 5% BSA for an hour, the cells were incubated at 4 °C overnight with primary antibodies (anti-BTN3A1 (1: 100, Abcam ab236289 Cambridge, UK) and anti- HIF-1α (1: 100, CST 79233 Danvers, MA, USA) or anti-BTN3A1 and anti-ULK1 (1: 50, Santa Cruz sc-390904 California, USA)).Fluorescein isothiocyanate-conjugated secondary antibody was used to incubated with the cells for 60 min at room temperature. After rinses with PBS, the cells were counterstained with 4’,6-Diamidino-2-phenylindole (DAPI) (Solarbio C0065, Beijing, China) for 15 min in the dark. Images of five randomly selected fields were captured with an Olympus DP72 microscope, and fluorescence density was calculated with Image J 1.48 V software.

The mRFP-GFP-LC3 lentivirus vectors were used to monitor autophagy flux (GeneChem, Shanghai, China). Transfected cells were selected with puromycin. Cells were grown on coverslips and fixed for 20 min with 4% paraformaldehyde at room temperature. Images were obtained using a confocal laser scanning microscope (Zeiss LSM 880). Three random fields were selected for puncta quantification. Vector information is listed in related manuscript file.

### Transmission electron microscopy

Cells were collected, washed twice with PBS and fixed with 2.5% glutaraldehyde for 24 h. Subsequently, the samples were dehydrated, embedded, and stained with uranyl acetate/lead citrate. Images were captured using a transmission electron microscope (JEM-1400PLUS; JEOL Ltd, Japan).

### In vivo experiments

All animal procedures were performed according to the guidelines outlined by the Institutional Animal Care and Use Committee of Shandong University and were approved by the ethics committee of Qilu Hospital. Five-week-old BALB/c-nude male mice were purchased from Beijing Sibeifu Biotechnology (Beijing, China). Twenty nude male mice were randomly assigned to four groups (*n* = 5 per group): (1) BTN-NC, (2) BTN-OE, (3) shNC, and (4) shBTN. Single-cell suspensions of 1 × 10^7^ cells were injected subcutaneously into the axilla. Tumors were measured twice a week, and the tumor volume was evaluated using the following formula: total tumor volume (mm^3^)=0.5^2^ × length × width^2^. Twenty mice were randomly assigned to four groups: (1) control shRNA, (2) BTN3A1 shRNA, (3) IR plus control shRNA, and (4) IR plus BTN3A1 shRNA. KYSE150 cells (1 × 10^7^) carrying the corresponding vector were injected subcutaneously into the axilla of the mice. The tumor volume (V) was measured every 3 days and was calculated using the following formula: *V* = (*ab*^2^)/2 (*a* is the long diameter and *b* is the short diameter). When tumors reached a volume of ~200 mm^3^, radiation was administered at a dose of 4 Gy on days 1, 3, and 5 in the specific tumor area. Tumor sizes were measured for every 3 days for ~3 weeks. Then, the mice were sacrificed. Tumors were removed and collected for immunostaining analyses.

### Immunohistochemistry and staining analysis

Paraffin-embedded esophageal cancer tissues were sectioned at a thickness of 4 μm. After an incubation at 65 °C for 2 h, the tissue sections were deparaffinized and rehydrated. Sections were heated at 95 °C in 0.01 M citrate buffer (pH = 6.0). Normal goat serum was used to block the tissue. The tissues were incubated with a primary antibody at 4 °C overnight. The primary antibody was an anti-BTN3A1 antibody (1:100, Abcam, ab236289) or anti-LC3B antibody (1:100, Abcam, ab48394). Subsequently, the sections were incubated with a secondary antibody for 1 h and stained with DAB (Zhongshan Biotech, Beijing, China). Hematoxylin counterstaining was completed, and all the sections were dehydrated and sealed.

Two experienced pathologists independently evaluated the percentage of positive tumor cells and their staining intensity [42]. According to the semi-quantitative system, the values of BTN3A1 and LC3B staining intensity were assigned as follows: 0 (no staining), 1 (weak), 2 (moderate), and 3 (strong). The percentage of positive tumor cells was classified as follows: 0 (0–5%), 1 (5–25%), 2 (26–50%), 3 (51–75%), and 4 (76–100%). The final score was determined by multiplying the values of these two scores. According to the score, the samples were divided into a BTN3A1 low expression group (score <6 points) and a BTN3A1 high- expression group (score of 6–12 points).

### Coimmunoprecipitation, mass spectrometry, and western blot

Cells were lysed after transfection with the designated plasmids in IP lysis buffer (Beyotime Biotechnology, USA) containing protease inhibitor cocktail (Roche). Extracted proteins were measured by immunoprecipitation with special antibodies: an anti-ULK1 antibody (1:50 Cell Signaling Technology, 8054), anti-IgG (Beyotime, A7016, A7028), primary anti-Flag Magnetic beads (1:20 Beyotime, P2115), and Protein A/G PLUS-agarose beads (1:50 Santa Cruz Biotechnology, sc-2003). After washes with PBS to remove unbound proteins, the residual proteins were suspended in 1× SDS- sample buffer and boiled for 10 min. The eluted proteins were subjected to a mass spectrometry analysis using LC–MS/MS (Novogene-labs, China). IgG (Cell Signaling Technology, Danvers, MA) was used as a negative control. The results of the mass spectrometry data were uploaded to https://www.iprox.org/. Specific proteins were visualized using an Immobilon™ chemiluminescence WB detection system (Merk Millipore, Germany). WB analyses were performed as previously described [43]. The primary antibodies are listed in Supplementary Table S9.

### Chromatin immunoprecipitation

A ChIP kit from Epigentek (Farmingdale, NY, USA) was used with a rabbit anti- HIF-1α primary antibody (5 µg for 10^5^ cells, Abcam, ab51608), rabbit IgG (Millipore, USA) as the negative control and anti-RNA Polymerase II (Epigentek) as the positive control. The ChIP products were added at a 1:20 dilution, subjected to RT‒PCR and separated on 1.5% agarose gels. BTN3A1 primers for ChIP assays were synthesized by Accurate Biotechnology (Hunan, China) and are listed in Supplementary Table S10.

### Luciferase reporter assay

The wild-type or mutant-type BTN3A1 promoter was sub-cloned into the pL01-Basic luciferase expression vector Gene Meditech (Shanghai, China). KYSE150 cells were plated into 24-well plates and transiently co-transfected with luciferase plasmids together with pM14 HIF-1α plasmids using DNA Transfection Reagent (jetPRIME). After 24 h transfection, cell lysate was harvested, and luciferase activity was measured using a Pair™ Duo-Luciferase Assay Kit 2.0. Firefly luciferase activity was normalized to the activity of the Renilla luciferase control. Vector information for pL01 and pM14 is listed in related manuscript file.

### Database analysis

BTN3A1 transcriptome data were downloaded from TCGA database (https://tcga-data.nci.nih.gov/tcga/) and Gene Expression Omnibus (GEO) database (Accession Number GSE161533, GES20347, and GSE17351) (https://www.ncbi.nlm.nih.gov/geo/). Autophagy related genes were downloaded from the HADb Human Autophagy database (http://www.autophagy.lu/clustering/index.html).

### Statistical analysis

Statistical analyses were conducted using SPSS version 21.0 (SPSS, Inc, Chicago, IL, USA) and GraphPad Prism 8.0 software (GraphPad Software, Inc., CA, USA). All data were analyzed for normality and equal variance. Continuous variables were presented as mean ± SD, and the data between two groups were analyzed using student’s t test or Mann–Whitney U test as appropriate. The associations of BTN3A1 expression with recurrence rate and clinicopathologic characteristics was determined using the Chi-square test. OS and RFS were evaluated by the Kaplan–Meier method and compared with the log-rank test. The predictive value of BTN3A1 expression in the specimen was determined by analyzing the ROC curve analysis and estimating the AUC. A univariable Cox analysis was performed to determine the prognostic significance of the parameters. Multivariate Cox proportional-hazard model was used to evaluate the independent prognostic factors and estimate 95% confidence intervals (CIs). The parametric generalized linear model with random effects was performed for cell growth and the CCK8 assay, whereas the single-hit multitarget model was used for colony formation assay. X-tile 3.6 software (Yale University School of Medicine, New Haven, USA) was used to analyze the immunohistochemical score, and Pearson’s correlation tests were conducted to analyze the correlation of BTN3A1 and LC3B. No statistical method was used to calculate the sample size, and no sample values were excluded during analysis. Single, double, and triple asterisks indicate statistical significance (*p < 0.05, **p < 0.01, and ***p < 0.001).

## Supplementary information

Supplementary Fig.S1

Supplementary Fig.S2

Supplementary Fig.S3

Supplementary Fig.S4

Supplementary Fig.S5

Supplementary Fig.S6

Supplementary Figure Legends

Supplementary Table S1

Supplementary Table S2

Supplementary Table S3

Supplementary Table S4

Supplementary Table S5

Supplementary Table S6

Supplementary Table S7

Supplementary Table S8

Supplementary Table S9

Supplementary Table S10

aj-checklist

Uncropped Western blots

Author Contribution Statement

Related Manuscript Information: Vector backbone information

Cell Line Authentication - STR Profiling Report

AJE Editing Certificate

Patient related data1

Patient related data2

## Data Availability

The datasets generated and/or analyzed during the current study are available from the corresponding author on reasonable request.
