# Supplementary figures and images for: BTN3A1 promotes tumor progression and radiation resistance in esophageal squamous cell carcinoma by regulating ULK1-mediated autophagy
Source: Cell Death Dis. 2022 Nov 22;13(11):984. doi: 10.1038/s41419-022-05429-w (PMC9684582; doi:10.1038/s41419-022-05429-w)

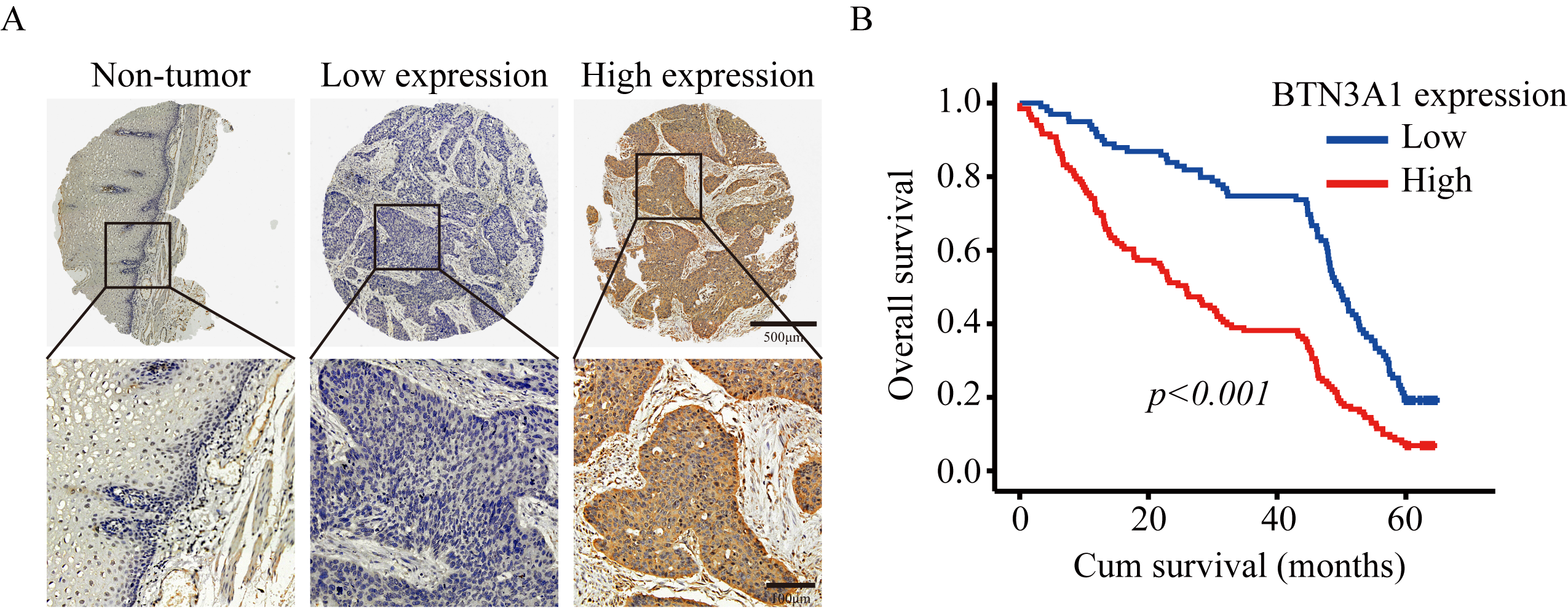

Supplement: Supplementary file 1 — Supplementary Fig.S1 [file 41419_2022_5429_MOESM1_ESM.tif]

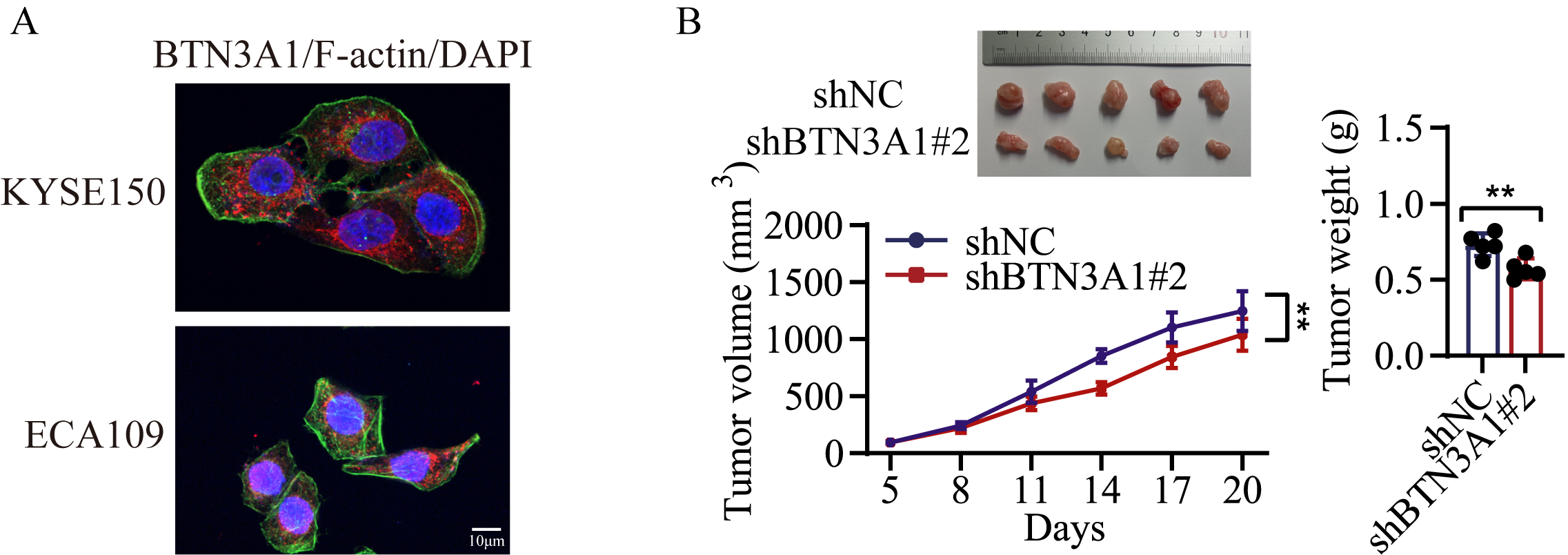

Supplement: Supplementary file 2 — Supplementary Fig.S2 [file 41419_2022_5429_MOESM2_ESM.tif]

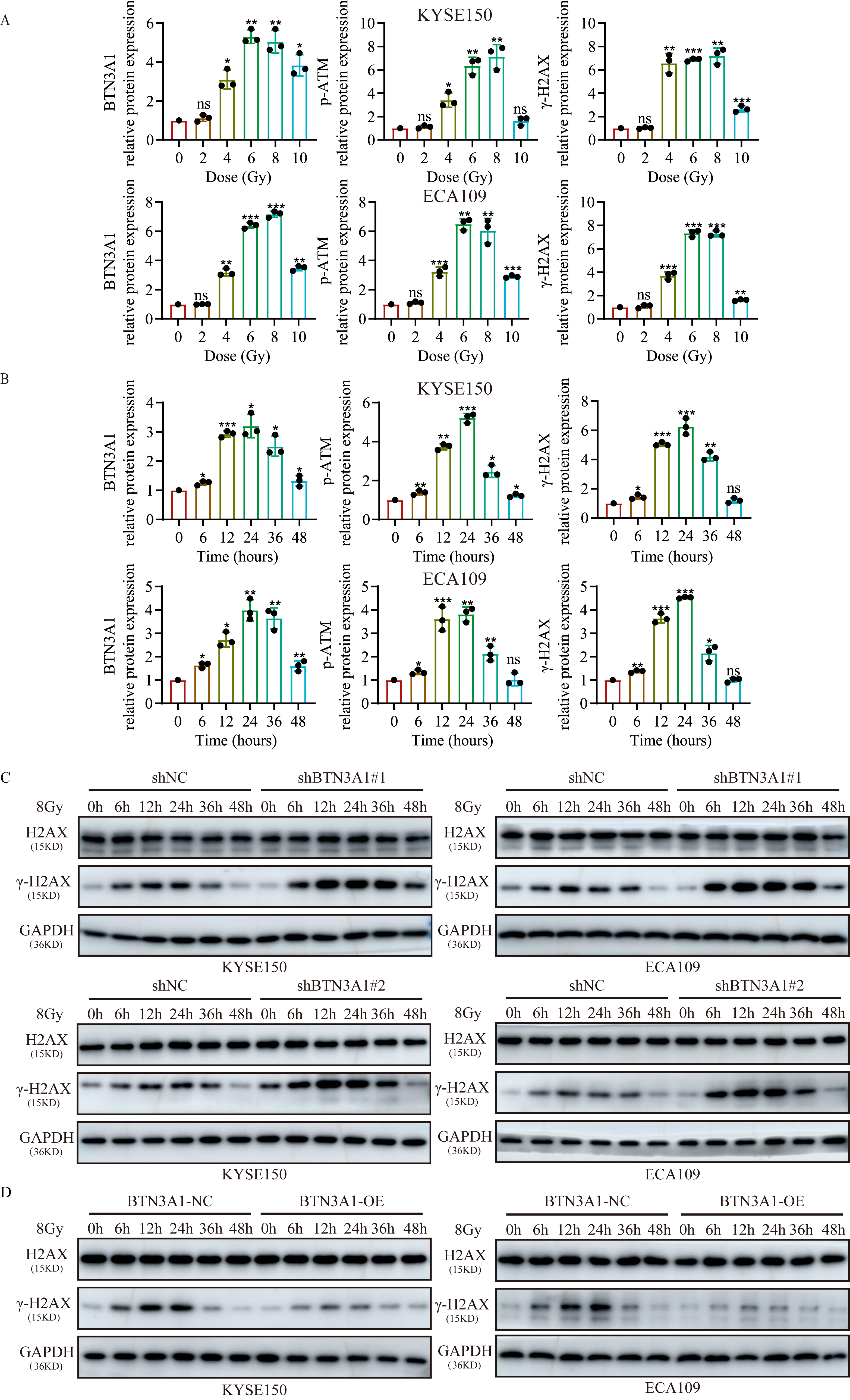

Supplement: Supplementary file 3 — Supplementary Fig.S3 [file 41419_2022_5429_MOESM3_ESM.tif]

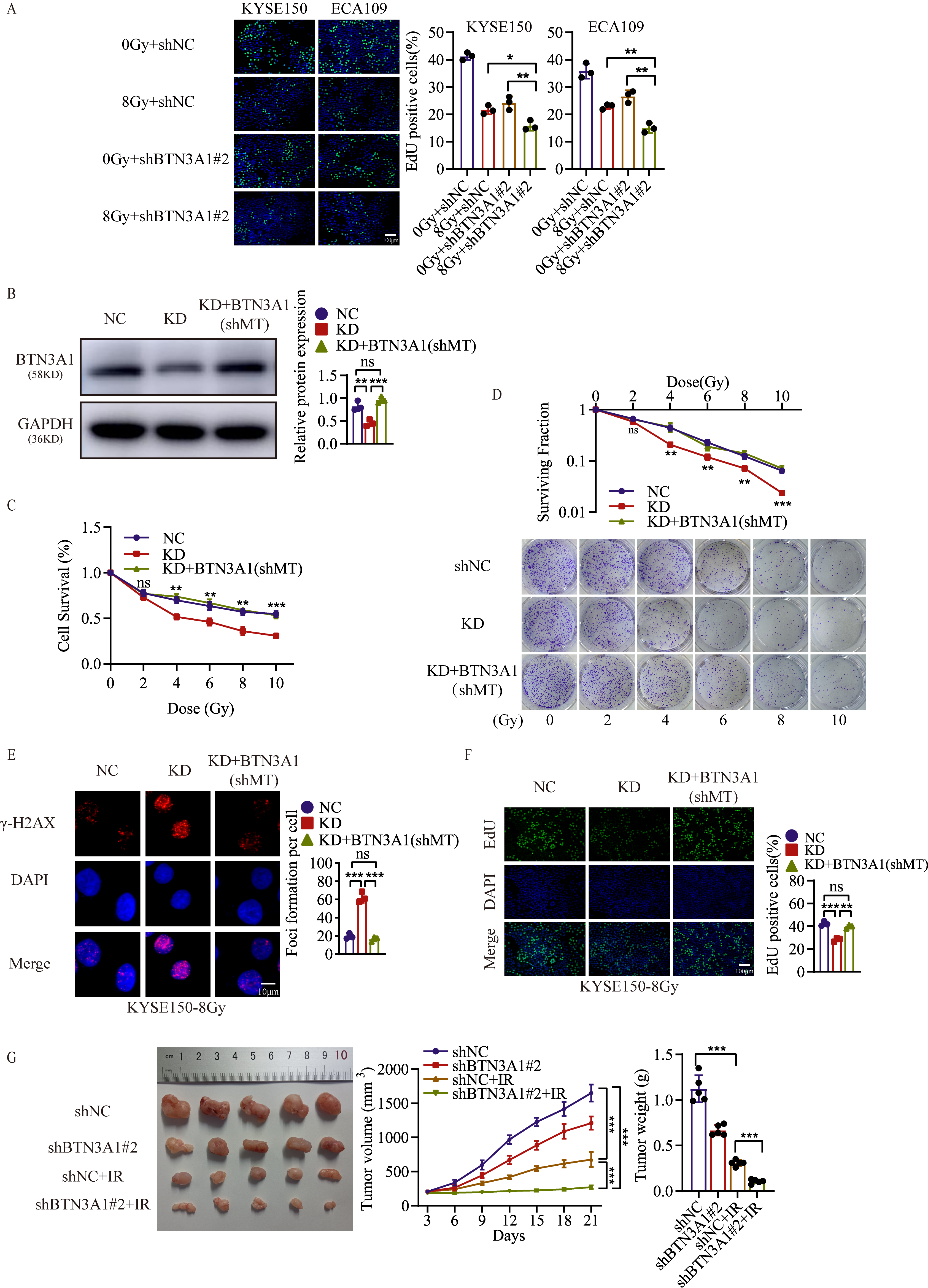

Supplement: Supplementary file 4 — Supplementary Fig.S4 [file 41419_2022_5429_MOESM4_ESM.tif]

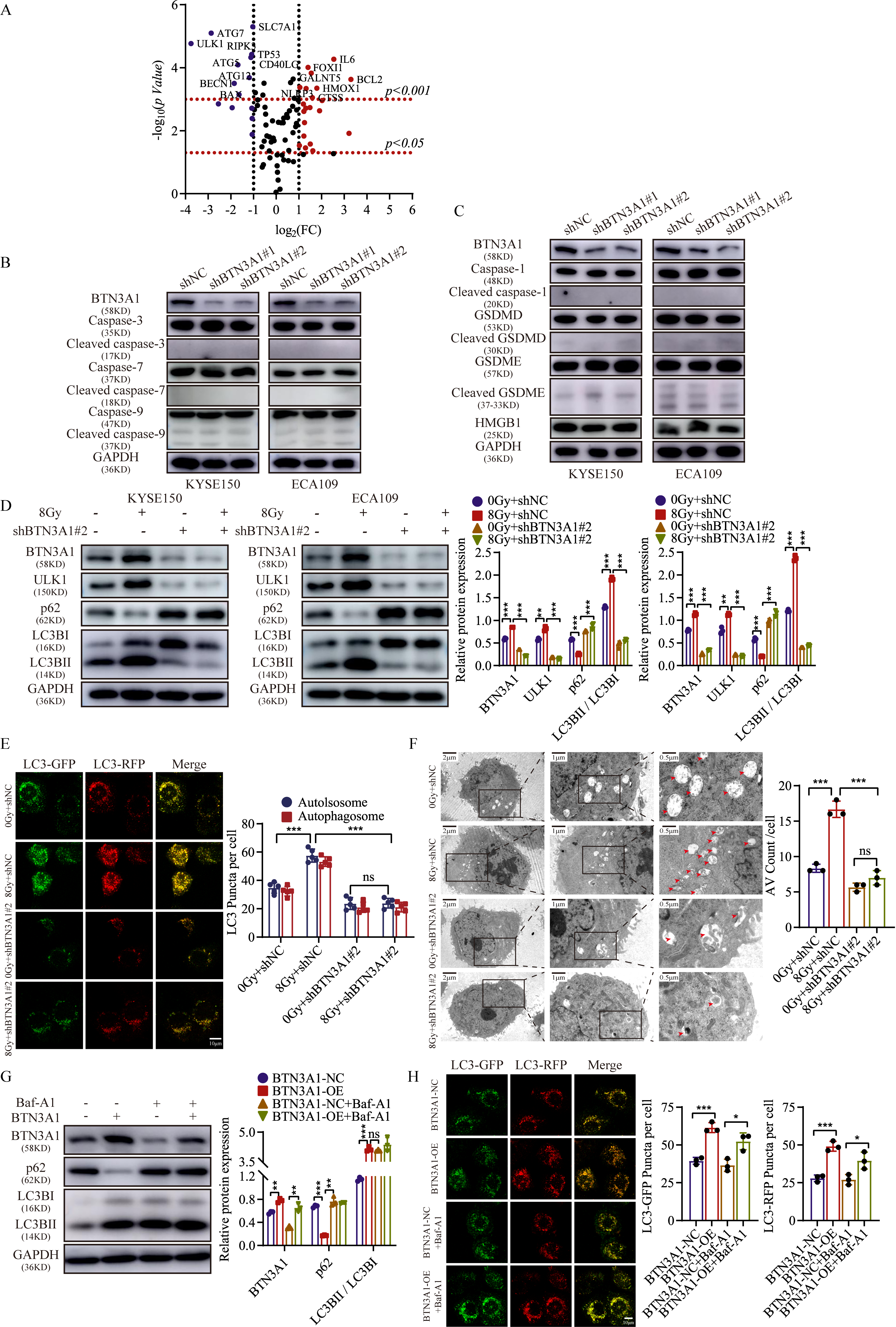

Supplement: Supplementary file 5 — Supplementary Fig.S5 [file 41419_2022_5429_MOESM5_ESM.tif]

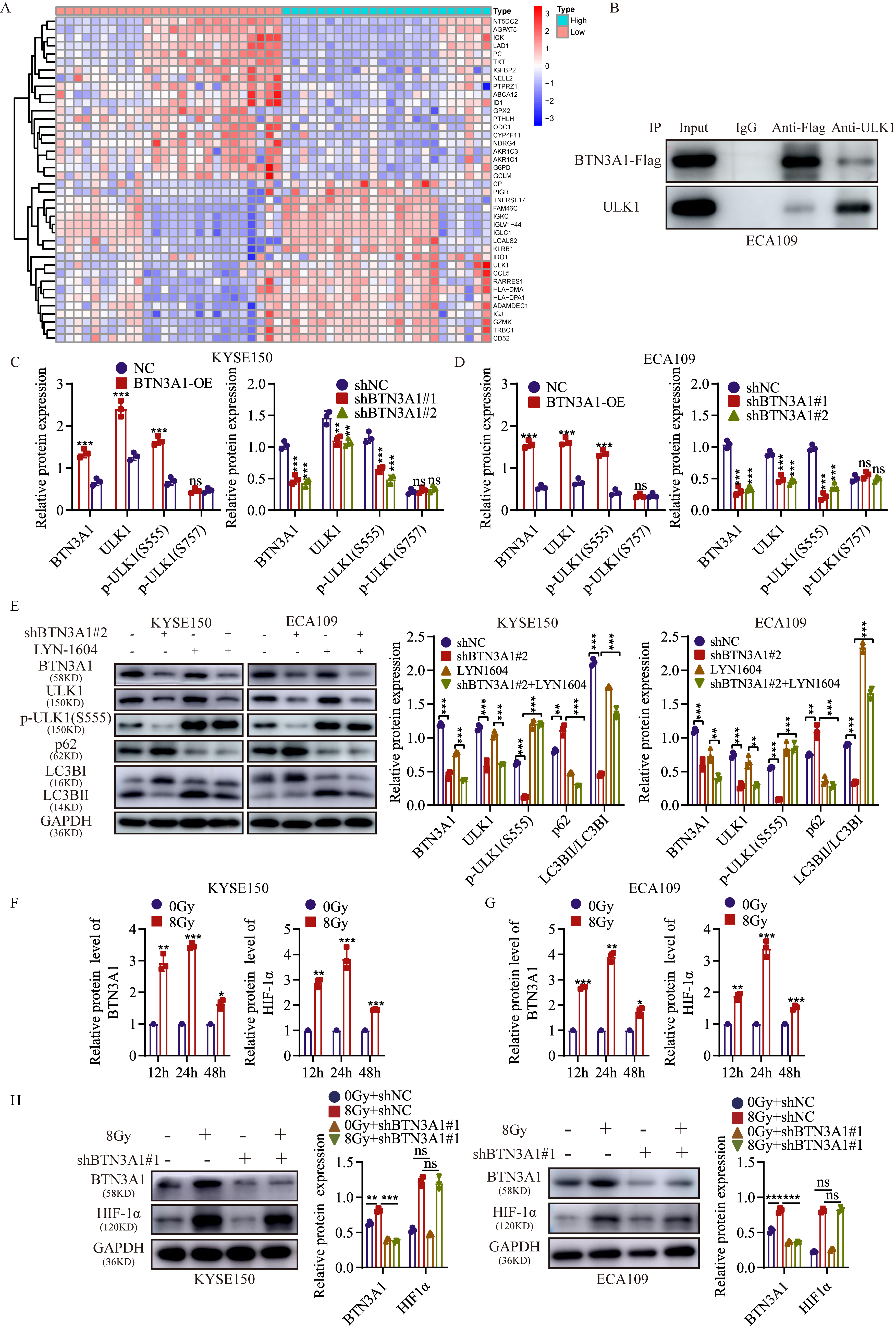

Supplement: Supplementary file 6 — Supplementary Fig.S6 [file 41419_2022_5429_MOESM6_ESM.tif]
