# Supplementary material for: BTN3A1 promotes tumor progression and radiation resistance in esophageal squamous cell carcinoma by regulating ULK1-mediated autophagy
Source: Cell Death Dis. 2022 Nov 22;13(11):984. doi: 10.1038/s41419-022-05429-w (PMC9684582; doi:10.1038/s41419-022-05429-w)
Supplement: Supplementary file 7 — Supplementary Figure Legends [file 41419_2022_5429_MOESM7_ESM.docx]

**Supplementary figure legends:**

**Supplementary Fig. S1.** (**A**) BTN3A1 is expressed at high levels in ESCC tumors and correlates with a poor prognosis for patients with ESCC. Representative images of different levels of BTN3A1 staining intensity: negative, weak and strong. (**B**) The OS curve indicates that BTN3A1 overexpression correlates with shorter OS.

**Supplementary Fig. S2.** (**A**) BTN3A1 is located in the cytoplasm of ESCC cells. IF staining showing BTN3A1 localization in KYSE150 and ECA109 cells. The nuclei were stained with 1 μg/ml DAPI (blue). The cytoskeleton was stained with 1μg/ml F-actin (red). Scale bar, 10 µm. (**B**) Transplanted xenografts derived from KYSE150 cells transfected with shBTN3A1#2 and shNC lentivirus were established in BALB/c nude mice (n=5). Tumor volumes and weights were measured.

**Supplementary Fig. S3.** (**A**) Statistical analysis of BTN3A1, p-ATM and γ-H2AX protein levels in cells after exposure to the different radiation doses (0-10 Gy). (**B**) Immunoblotting analysis of BTN3A1, p-ATM and γ-H2AX in cells collected at the indicated times after IR. These data are representative of three independent experiments. Means ± SD (n=3); ns, no significant difference; * *p* < 0.05;** *p* < 0.01, and *** *p* < 0.001. (**C and D**) The level of H2AX and γ-H2A.X protein in BTN3A1-KD (**C**) and BTN3A1-OE (**D**) cells treated with radiation (8Gy) in a time-point dependent manner.

**Supplementary Fig. S4.** (**A**) Effect of BTN3A1 knockdown on DNA synthesis. KYSE150 and ECA109 cells treated with or without radiation (8 Gy) were fluorescently stained with EdU (green). The nucleus was stained with Hoechst 33342 (blue). The percentage of EdU-positive cells was shown in the bottom panel. Scale bar: 100 µm. (**B**) BTN3A1 (shMT), a synonymous mutation, was constructed and transfected into KYSE150 cells to rescue BTN3A1 knockdown induced by transfecting a shRNA against BTN3A1 (sh-BTN3A1). WB analysis of BTN3A1 expression. (**C and D**) CCK-8 and colony formation assays were performed to evaluate cell survival when BTN3A1 expression was restored. (**E**) IF staining was used to examine γ-H2A.X foci. (**F**) EdU assays were conducted to evaluate DNA replication. These data are representative of three independent experiments. Means ± SD (n=3). (**G**) Representative images of xenografts from the indicated treatment groups. Tumor volumes and weights were measured. ns, no significant difference; * *p* < 0.05,** *p* < 0.01, and *** *p* < 0.001.

**Supplementary Fig. S5.** (**A**) Volcano plot showing the difference in the level and *p* value, which identified ULK1 as the most relevant gene. (**B and C**) Immunoblots of the key proteins involved in apoptosis (**B**) and pyroptosis (**C**) in cells with BTN3A1 knockdown. (**D**) Control and BTN3A1-knockdown KYSE150 and ECA109 cells were treated with IR (8 Gy) and cultured for 24 h, followed by immunoblotting with the indicated antibodies. (**E**) Control and shBTN3A1#2 KYSE150 cells were transfected with mRFP-GFP-LC3B and treated with or without radiation (8 Gy). The confocal microscopy analysis is shown. Scale bar, 10 µm. (**F**) Autophagosomes were observed using transmission electron microscopy. Red arrows indicated autophagosome formation. Scale bar, 2 µm. (**G**) Immunoblotting analysis of the protein levels of in KYSE150 cells stably overexpressing BTN3A1 and treated with 100 nM BafA1 for 24 h. (**H**) BTN3A1 increased the accumulation of mRFP-GFP-LC3B puncta in KYSE150 cells, but BafA1 inhibited the function of BTN3A1. Cells were transfected with NC or BTN3A1 [plasmid](file:///C:\Program%20Files%20(x86)\Youdao\Dict\7.5.1.0\resultui\dict\result.html?keyword=plasmid&lang=en) and treated with 100 nM BafA1 for 24 h. These data are representative of three independent experiments. Means± SD (n=3); ns, no significant difference; * *p* < 0.05;** *p* < 0.01, and *** *p* < 0.001.

**Supplementary Fig. S6.** (**A**) Heatmap of data from the HADb Human Autophagy database revealed that BTN3A1 expression was significantly correlated with ULK1 expression. The data were obtained from the ESCC samples in the GEO database (i.e., 50 ESCC cases, Accession Numbers GSE161533, GES20347, and GSE1735). (**B**) Coimmunoprecipitation of BTN3A1 and ULK1 was performed in ECA 109 cells. (**C and D**) Statistical analysis of BTN3A1, ULK1, p-ULK1(S555) and p-ULK1(S757) levels in KYSE150 and ECA109 cells transfected with BTN3A1-specific plasmid (BTN-OE), BTN3A1 shRNA lentivirus, or corresponding control.(**E**) Autophagy that was suppressed by shBTN3A1#2 was rescued by treatment with LYN-1604. (**F and G**) Statistical analysis of BTN3A1 and HIF-1α in KYSE150 and ECA109 cells with radiation (8 Gy) or not. The GAPDH protein level was used as the internal standard. The averages of triplicate experiments are shown. (**H**) KYSE150 or ECA109 cells were harvested 48 h after transfection with the shBTN3A1#1 lentivirus or corresponding control. The lysates were immunoblotted and quantified using ImageJ software. These data are representative of three independent experiments. Means ± SD (n=3); ns, no significant difference; * *p* < 0.05,** *p* < 0.01, and *** *p* < 0.001.
