# Supplementary material for: BTN3A1 promotes tumor progression and radiation resistance in esophageal squamous cell carcinoma by regulating ULK1-mediated autophagy
Source: Cell Death Dis. 2022 Nov 22;13(11):984. doi: 10.1038/s41419-022-05429-w (PMC9684582; doi:10.1038/s41419-022-05429-w)

Uncropped blots related to Figure2

Fig.2A

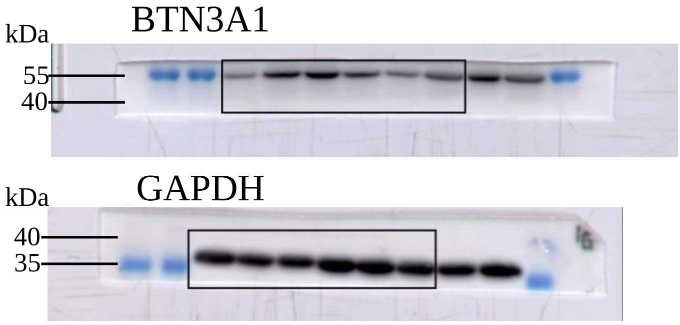

Fig.2B

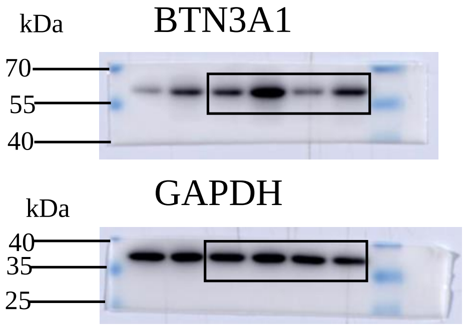

Fig.2C

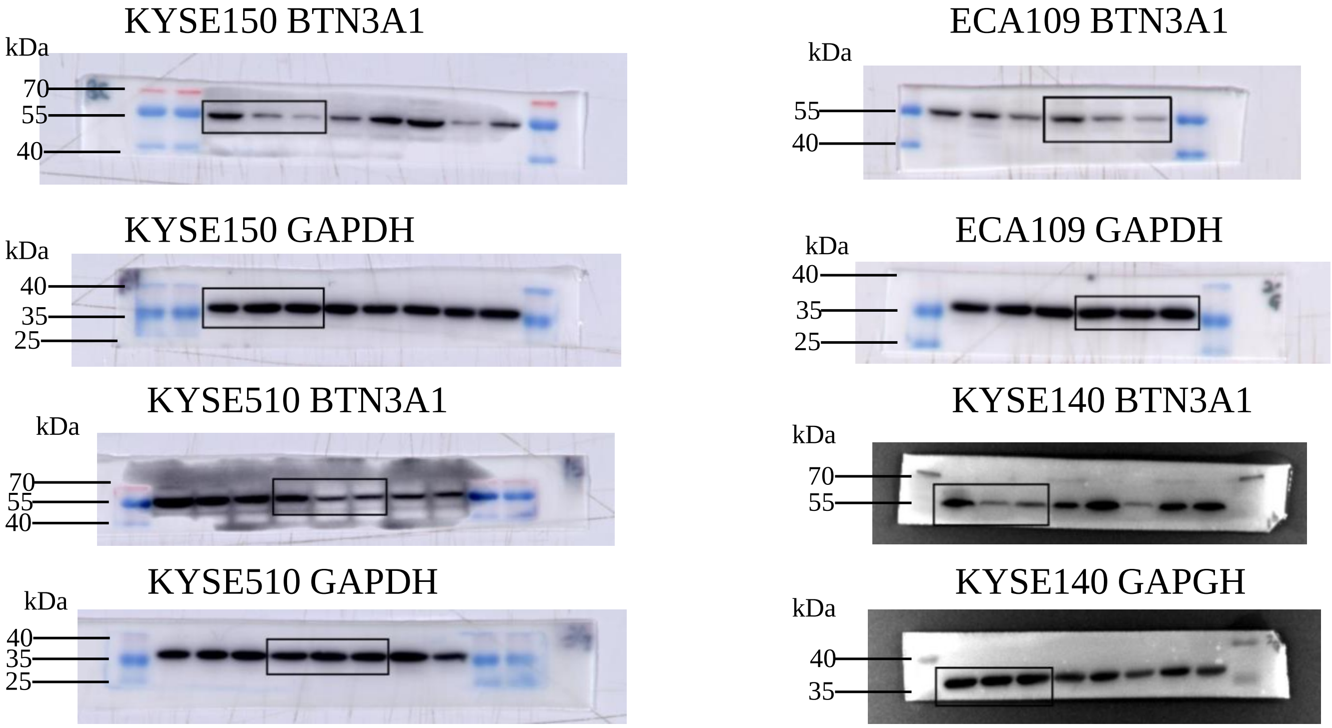

Uncropped blots related to Figure3

Fig.3A

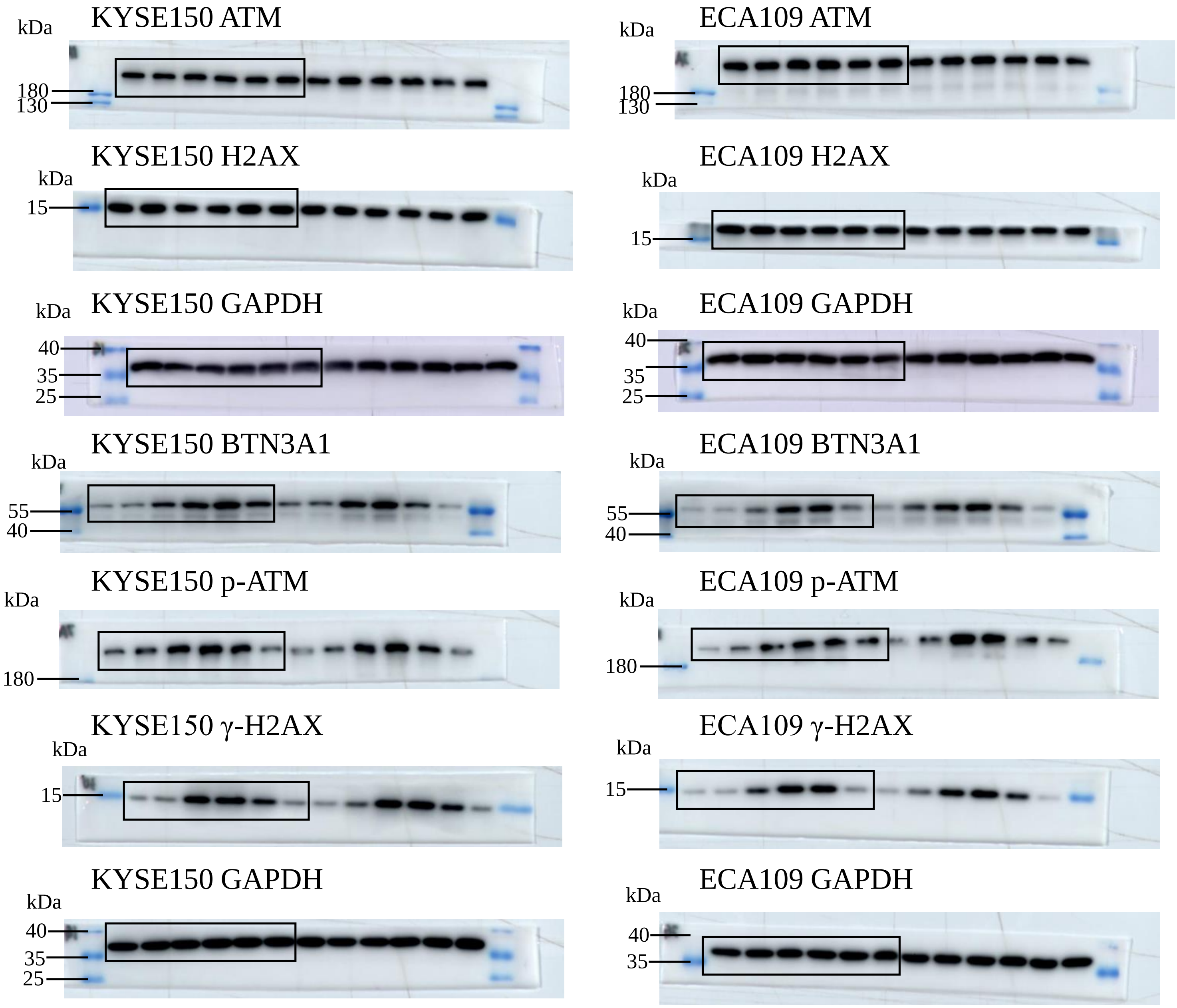

Uncropped blots related to Figure3

Fig.3B

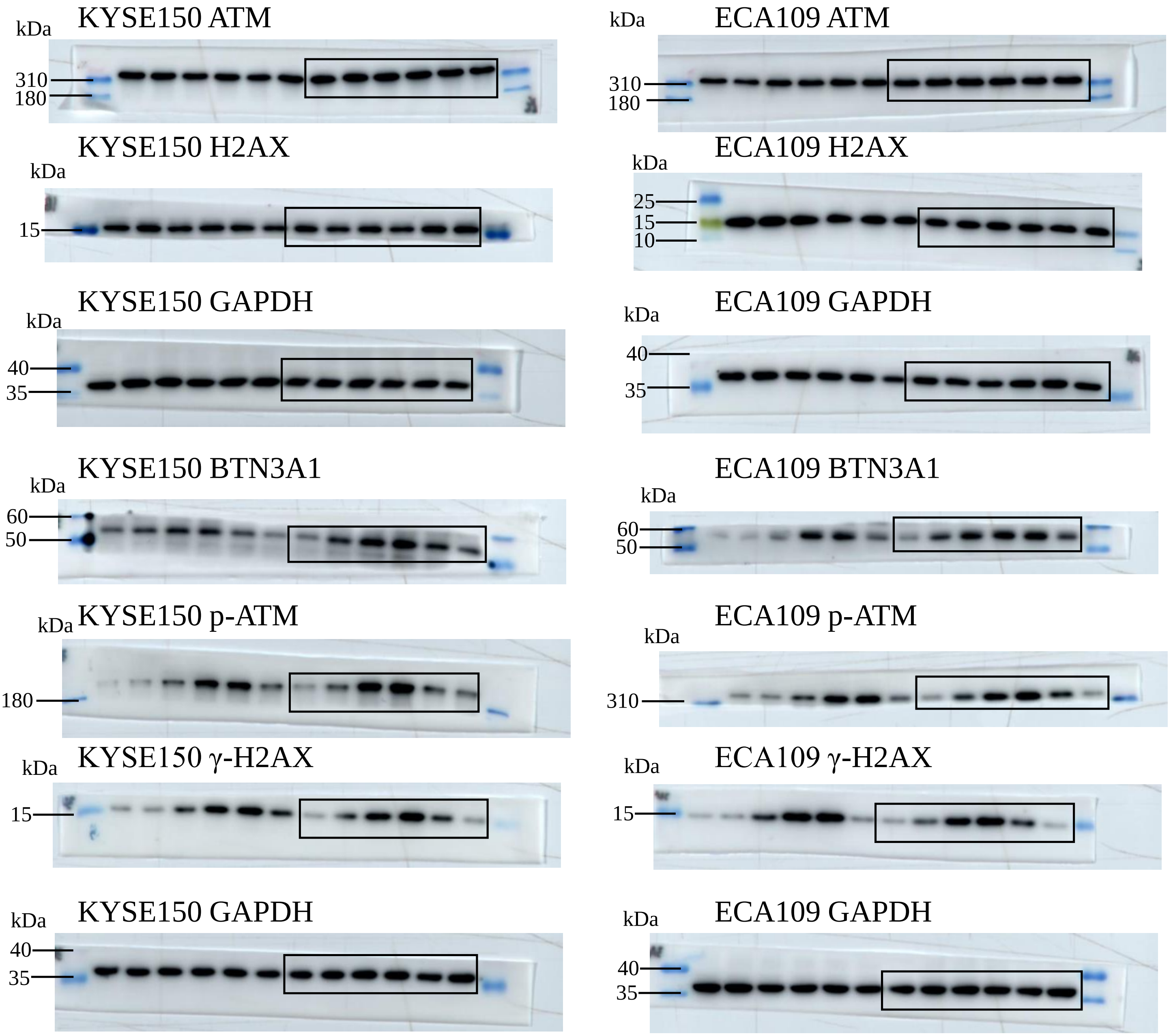

Uncropped blots related to Figure4

Fig.4B

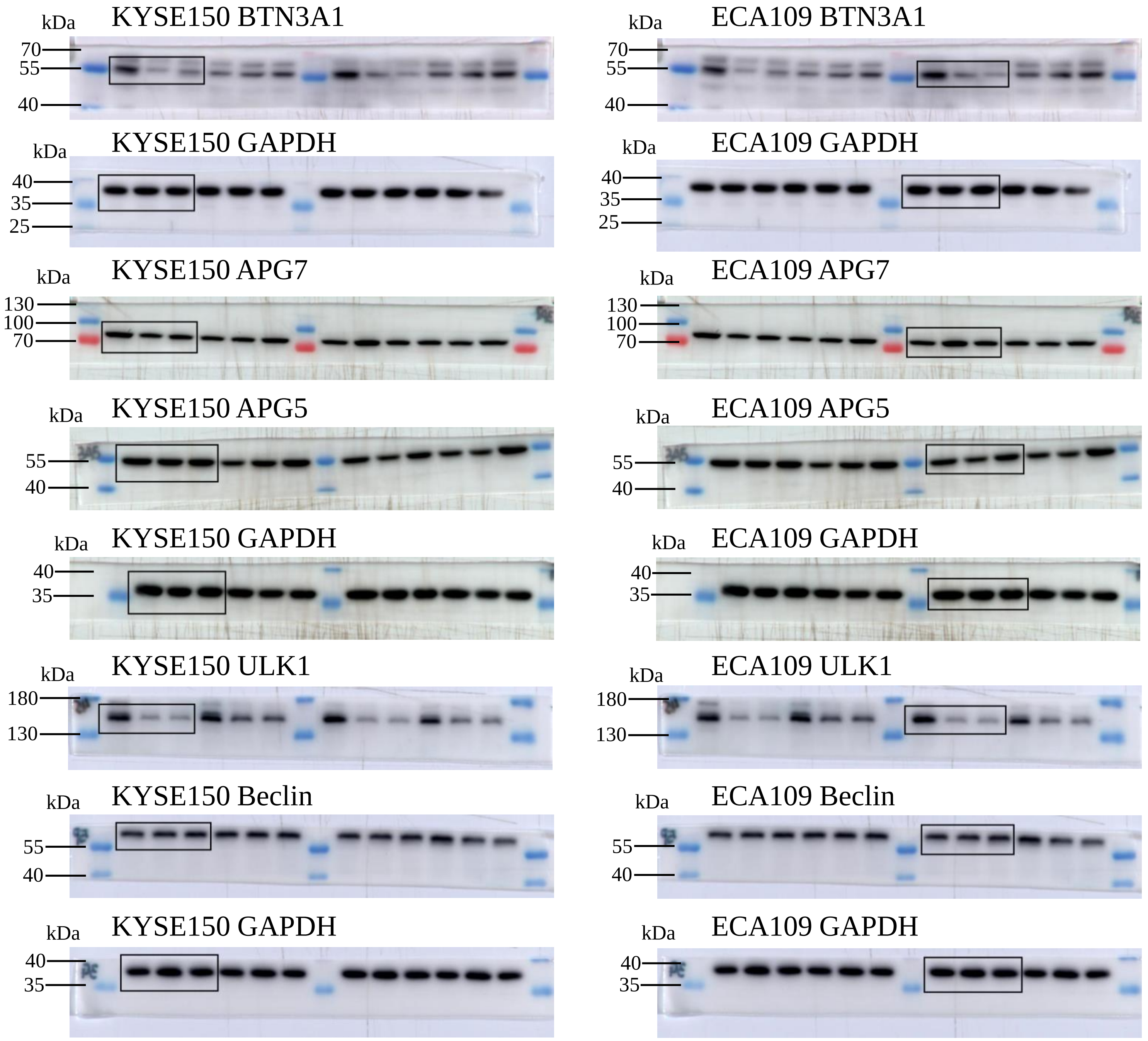

Fig.4B

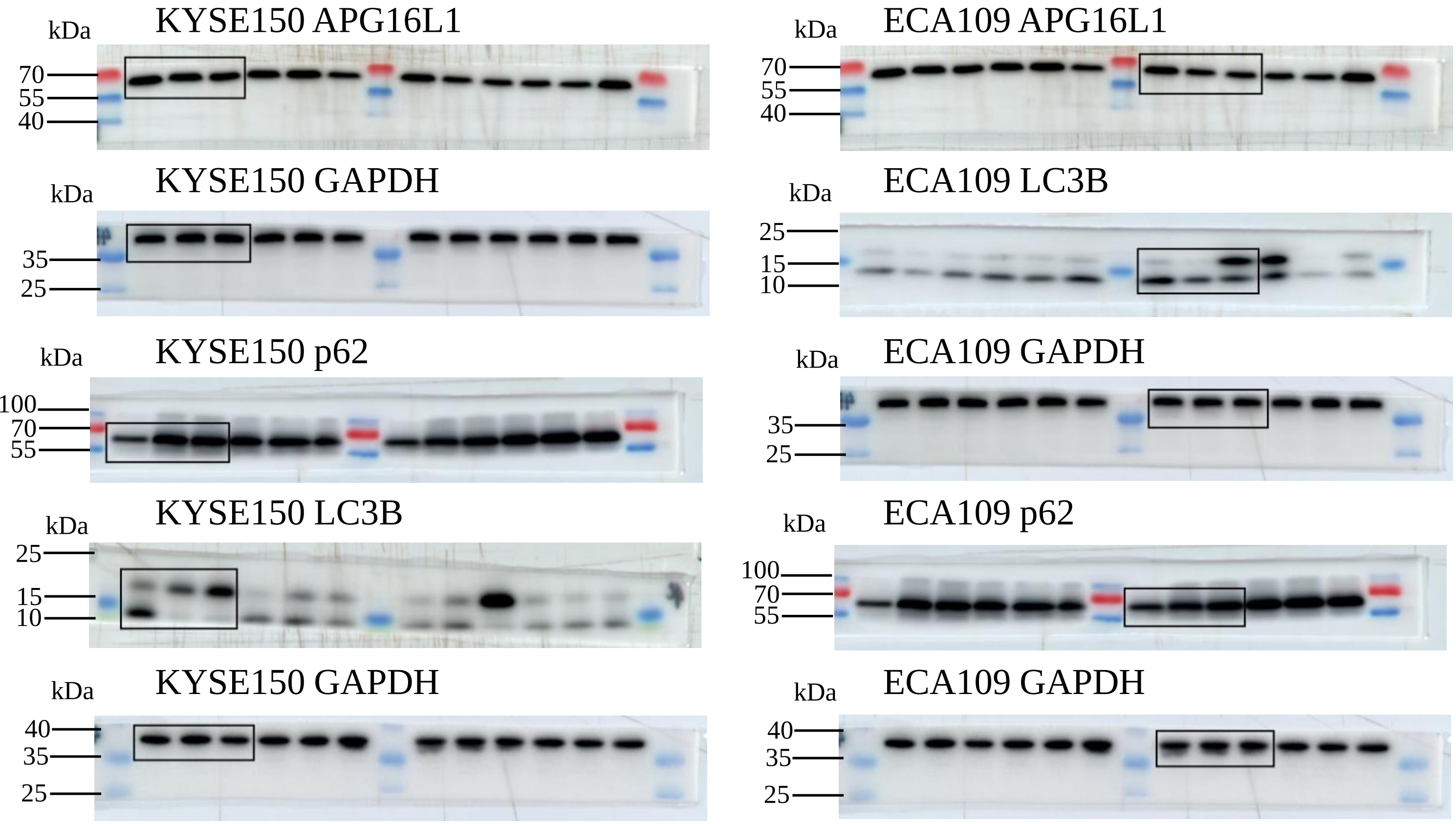
KYSE150 APG16L1
kDa
70
55
40
KYSE150 GAPDH
kDa
35
25
KYSE150 p62
kDa
100
70
55
KYSE150 LC3B
kDa
25
15
10
KYSE150 GAPDH
kDa
40
35
25
ECA109 APG16L1
kDa
70
55
40
ECA109 LC3B
kDa
25
15
10
ECA109 GAPDH
kDa
35
25
ECA109 p62
kDa
100
70
55
ECA109 GAPDH
kDa
40
35
25

Fig.4C

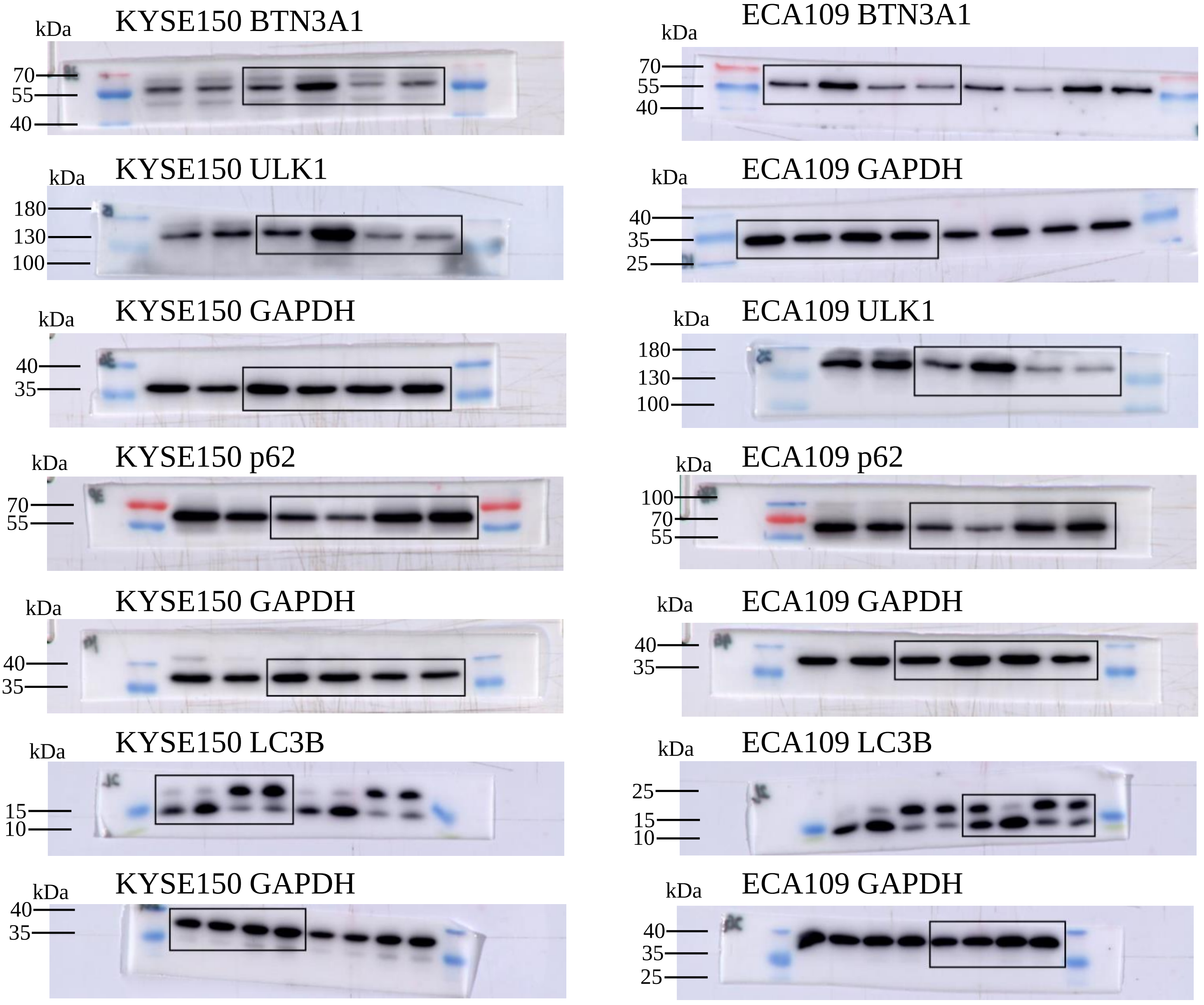
KYSE150 BTN3A1
kDa
70
55
40
ECA109 BTN3A1
kDa
70
55
40
KYSE150 ULK1
kDa
180
130
100
ECA109 GAPDH
kDa
40
35
25
KYSE150 GAPDH
kDa
40
35
ECA109 ULK1
kDa
180
130
100
KYSE150 p62
kDa
70
55
ECA109 p62
kDa
100
70
55
KYSE150 GAPDH
kDa
40
35
ECA109 GAPDH
kDa
40
35
KYSE150 LC3B
kDa
15
10
ECA109 LC3B
kDa
25
15
10
KYSE150 GAPDH
kDa
40
35
ECA109 GAPDH
kDa
40
35
25

Uncropped blots related to Figure6

Fig.6D

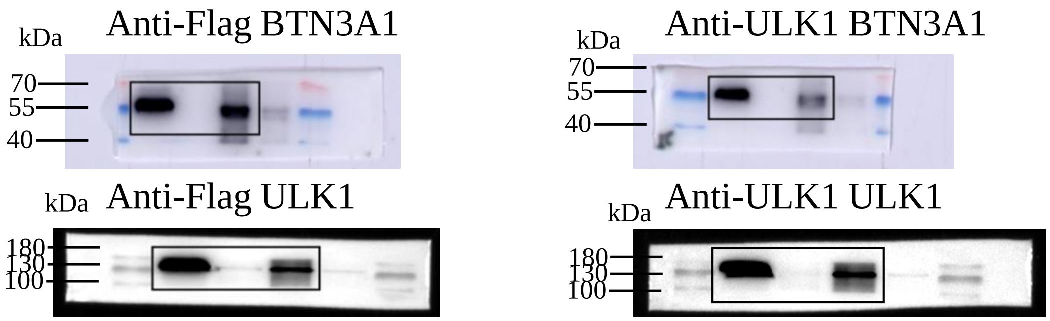

Fig.6E

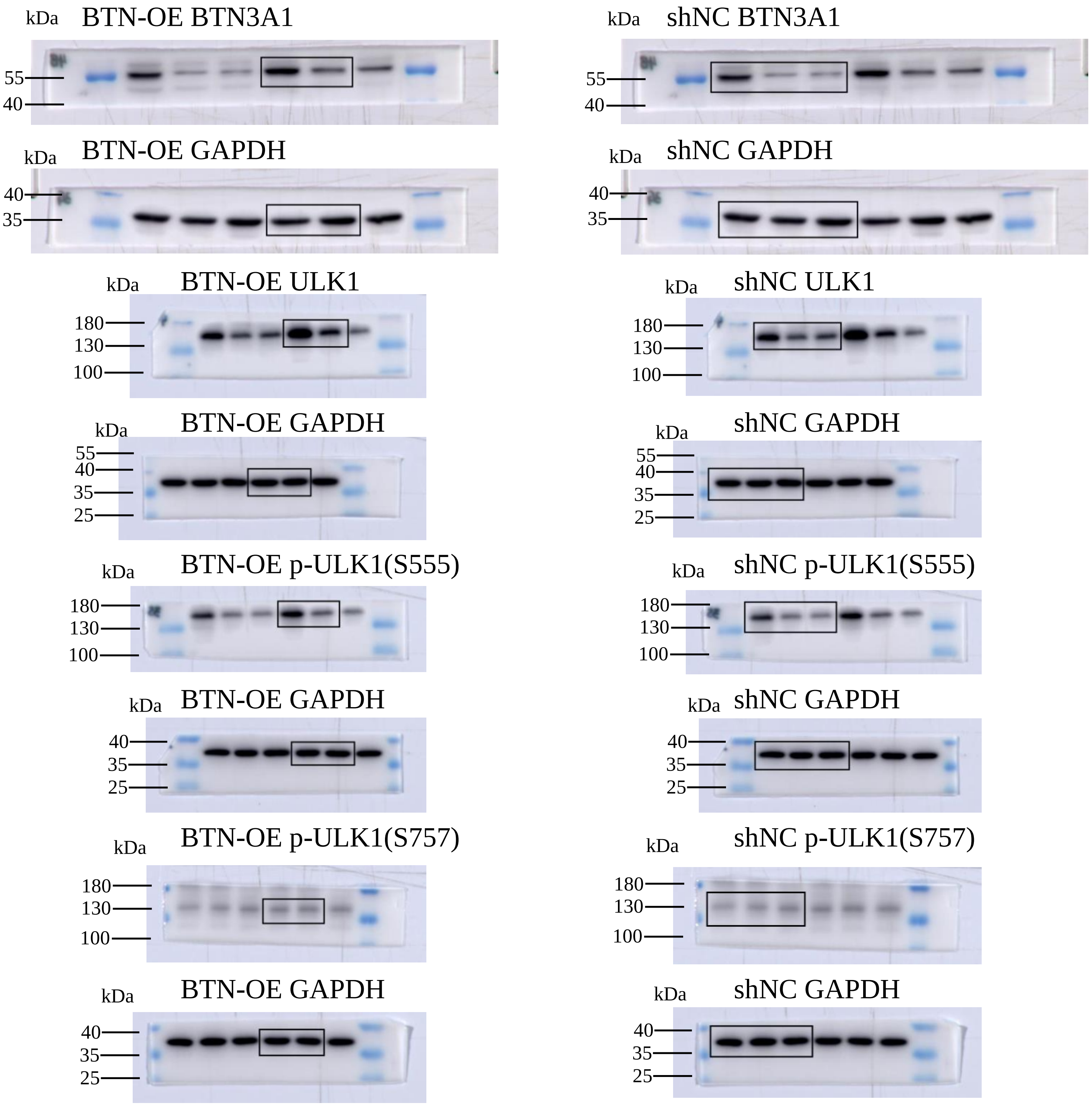

Fig.6F

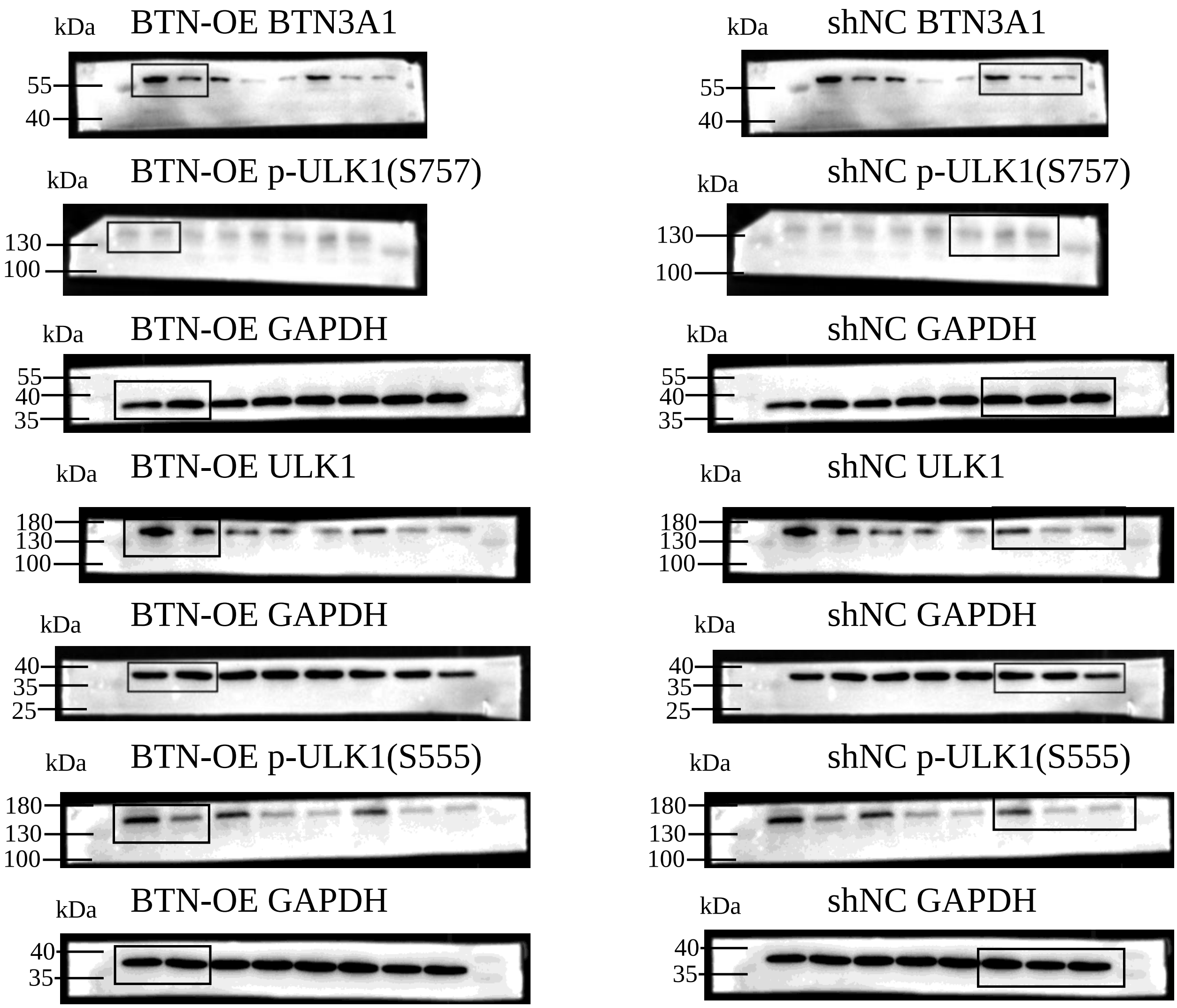
BTN-OE BTN3A1
shNC BTN3A1
BTN-OE p-ULK1(S757)
shNC p-ULK1(S757)
BTN-OE GAPDH
shNC GAPDH
BTN-OE ULK1
shNC ULK1
BTN-OE GAPDH
shNC GAPDH
BTN-OE p-ULK1(S555)
shNC p-ULK1(S555)
BTN-OE GAPDH
shNC GAPDH
kDa
180
130
100
55
40
35
25

Fig.6G

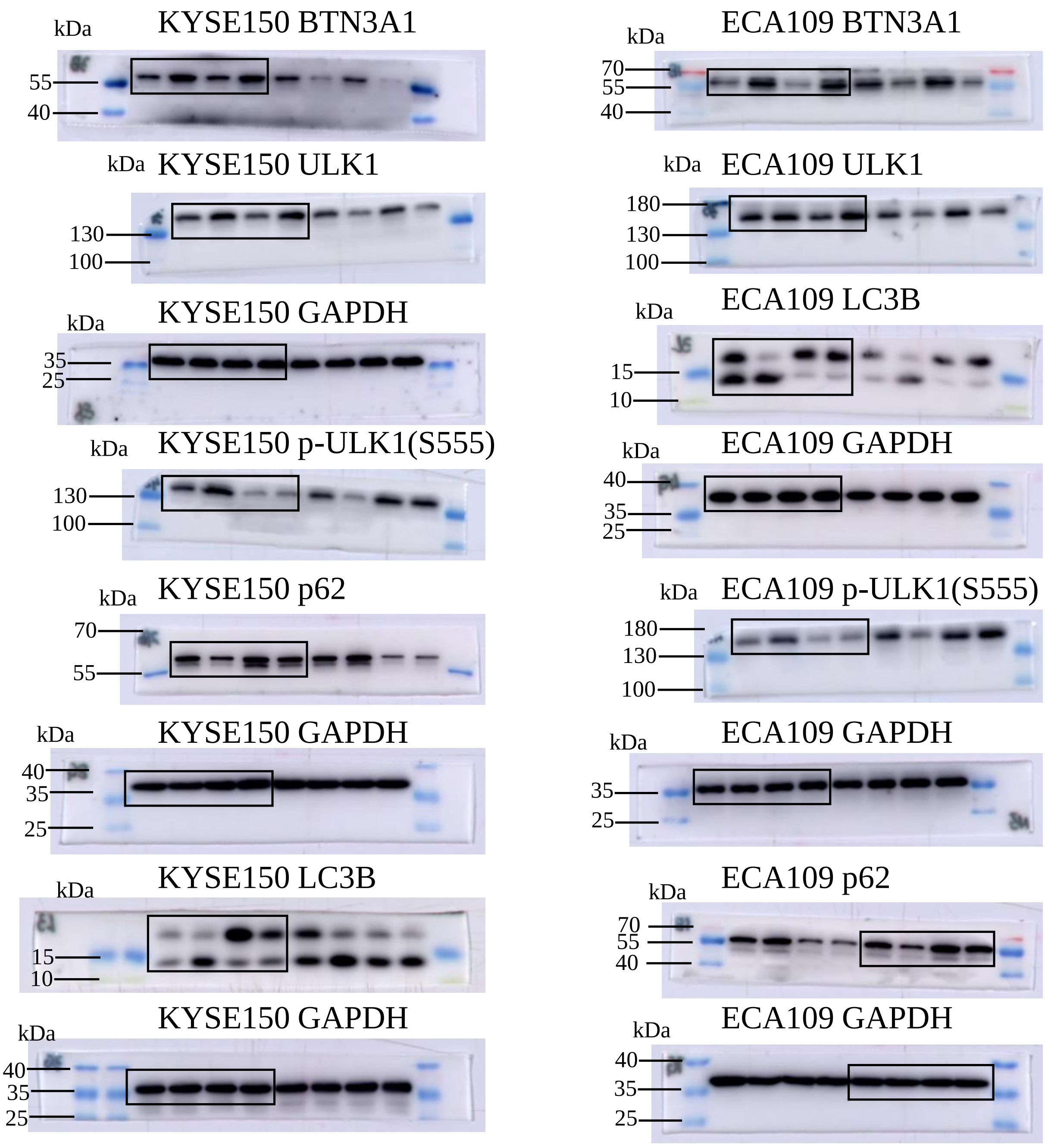
KYSE150 BTN3A1
kDa
55
40
KYSE150 ULK1
kDa
130
100
KYSE150 GAPDH
kDa
35
25
KYSE150 p-ULK1(S555)
kDa
130
100
KYSE150 p62
kDa
70
55
KYSE150 GAPDH
kDa
40
35
25
KYSE150 LC3B
kDa
15
10
KYSE150 GAPDH
kDa
40
35
25
ECA109 BTN3A1
kDa
70
55
40
ECA109 ULK1
kDa
180
130
100
ECA109 LC3B
kDa
15
10
ECA109 GAPDH
kDa
40
35
25
ECA109 p-ULK1(S555)
kDa
180
130
100
ECA109 GAPDH
kDa
35
25
ECA109 p62
kDa
70
55
40
ECA109 GAPDH
kDa
40
35
25

Fig.6H

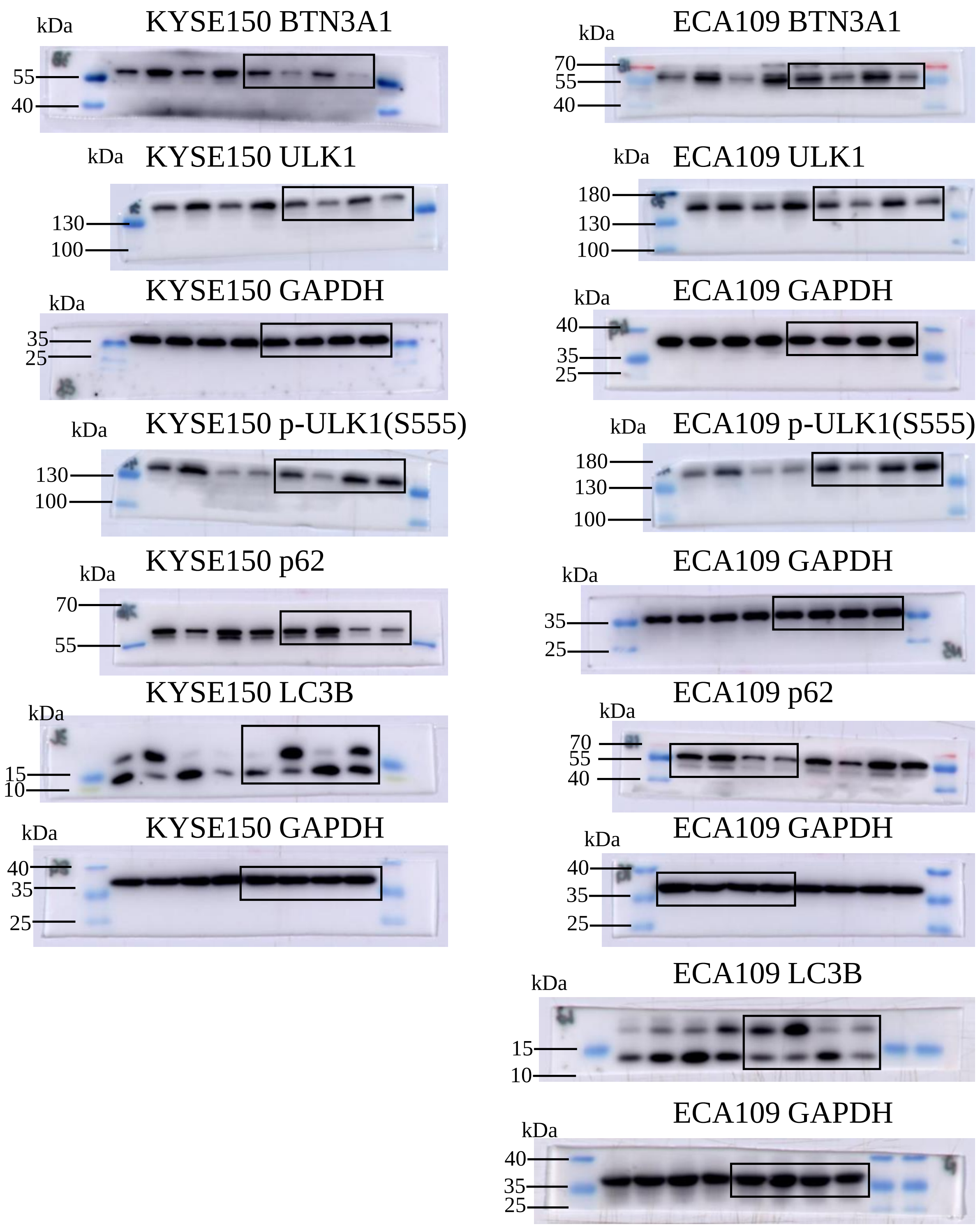
KYSE150 BTN3A1
kDa
55
40
ECA109 BTN3A1
kDa
70
55
40
KYSE150 ULK1
kDa
130
100
ECA109 ULK1
kDa
180
130
100
KYSE150 GAPDH
kDa
35
25
ECA109 GAPDH
kDa
40
35
25
KYSE150 p-ULK1(S555)
kDa
130
100
ECA109 p-ULK1(S555)
kDa
180
130
100
KYSE150 p62
kDa
70
55
ECA109 GAPDH
kDa
35
25
KYSE150 LC3B
kDa
15
10
ECA109 p62
kDa
70
55
40
KYSE150 GAPDH
kDa
40
35
25
ECA109 GAPDH
kDa
40
35
25
ECA109 LC3B
kDa
15
10
ECA109 GAPDH
kDa
40
35
25

Uncropped blots related to Figure7

Fig.7A

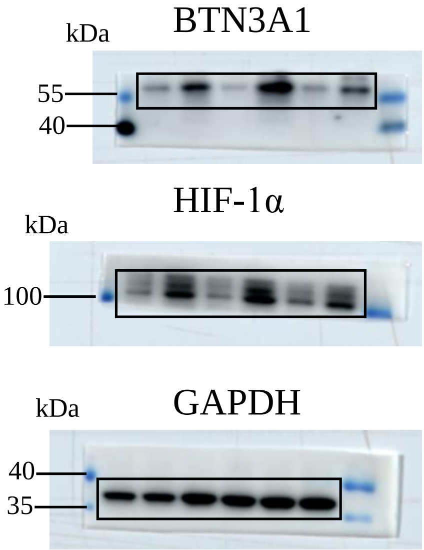

Fig.7B

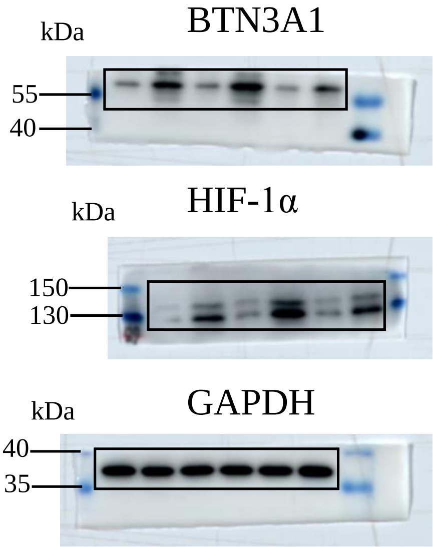

Fig.7C

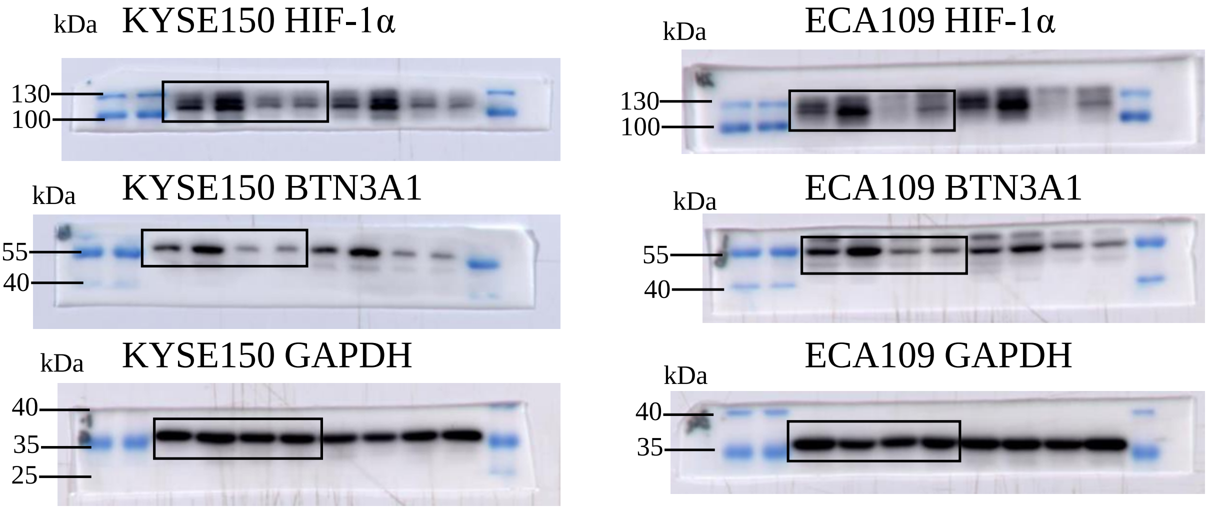

Fig.7D

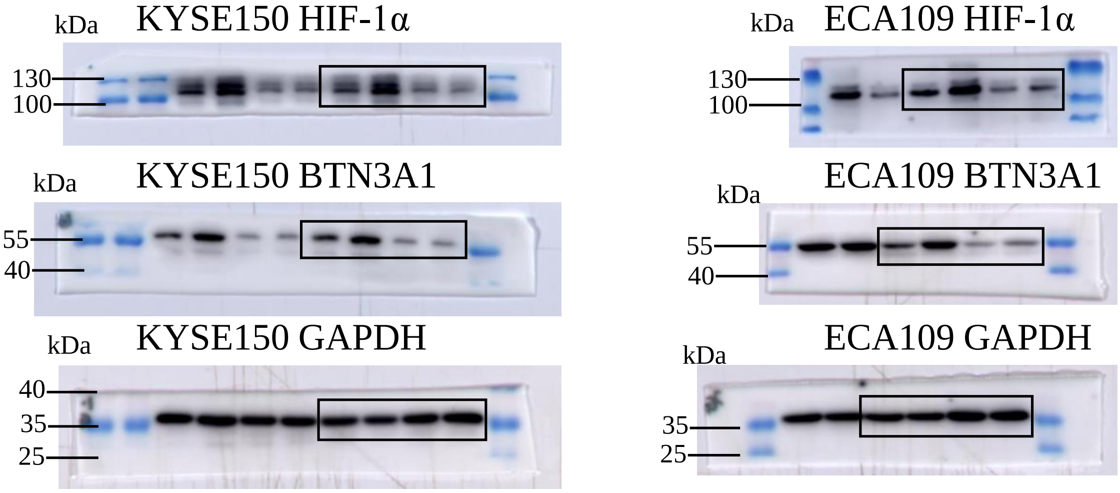

Uncropped blots related to Figure.S3&S4

Fig.S3C

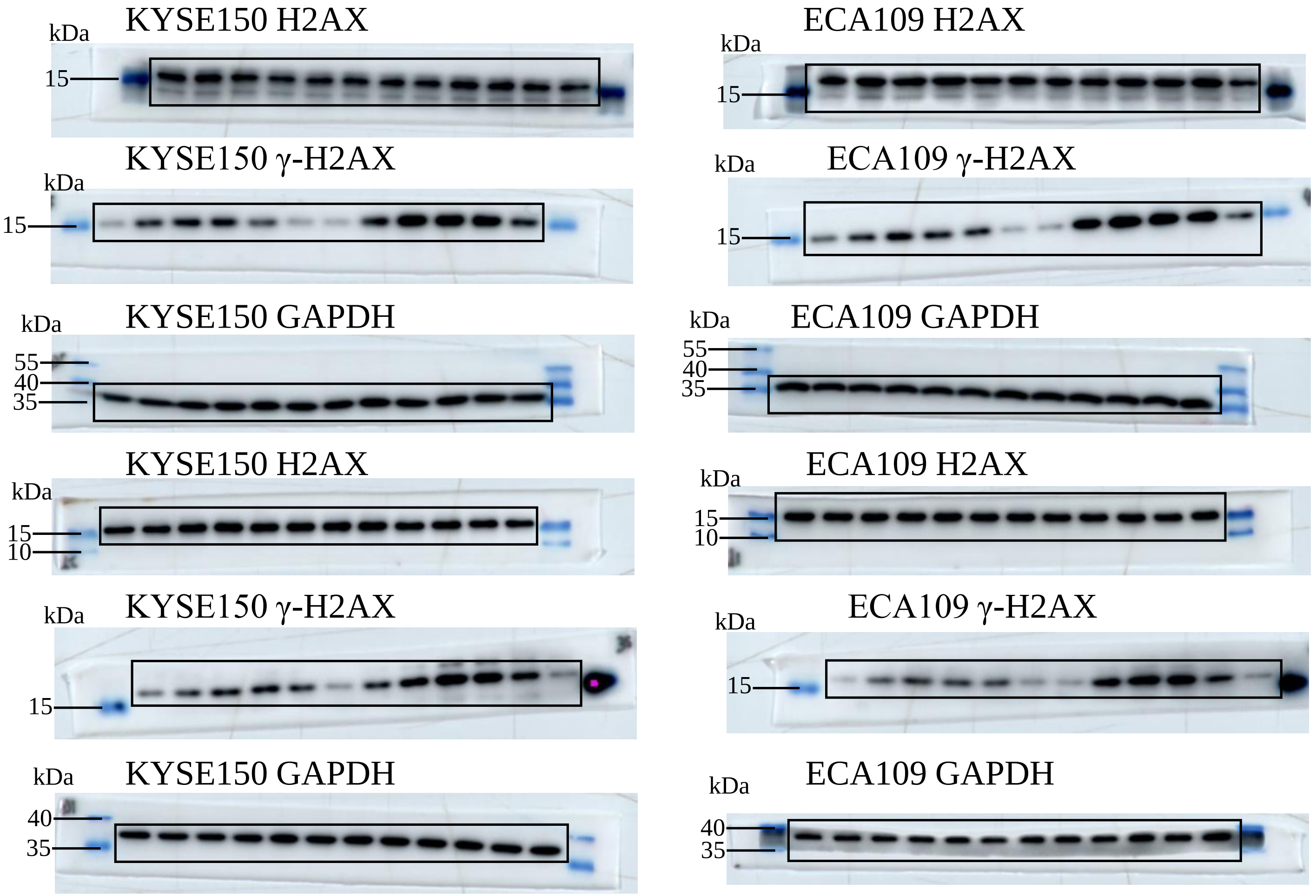

Fig.S3D

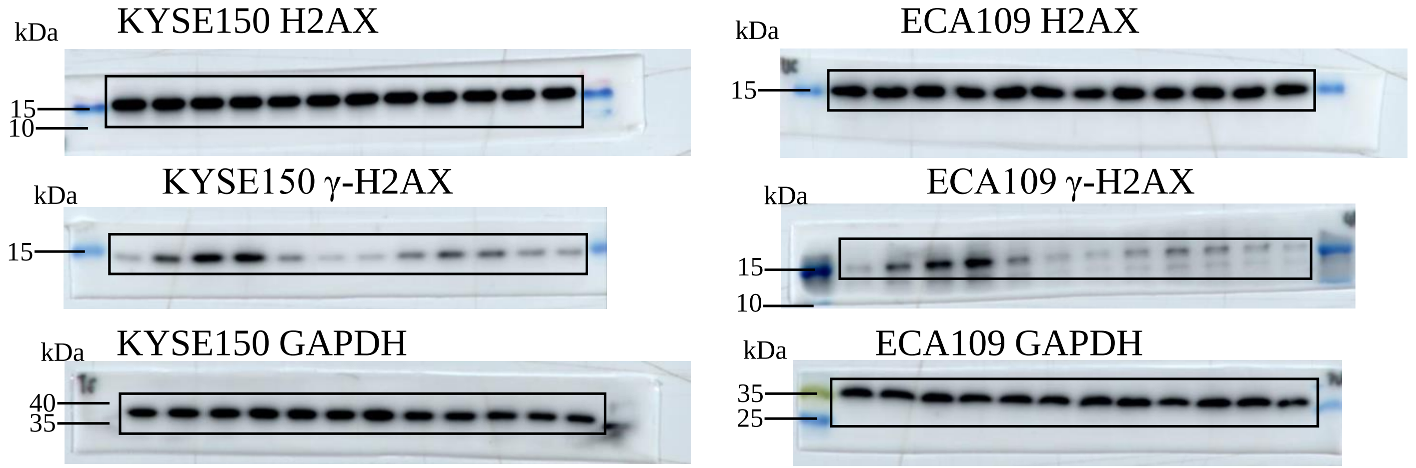

Fig.S4B

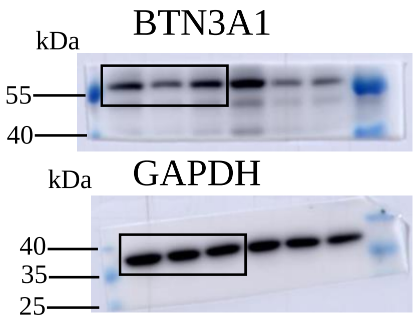

Uncropped blots related to Figure.S5

Fig.S5B

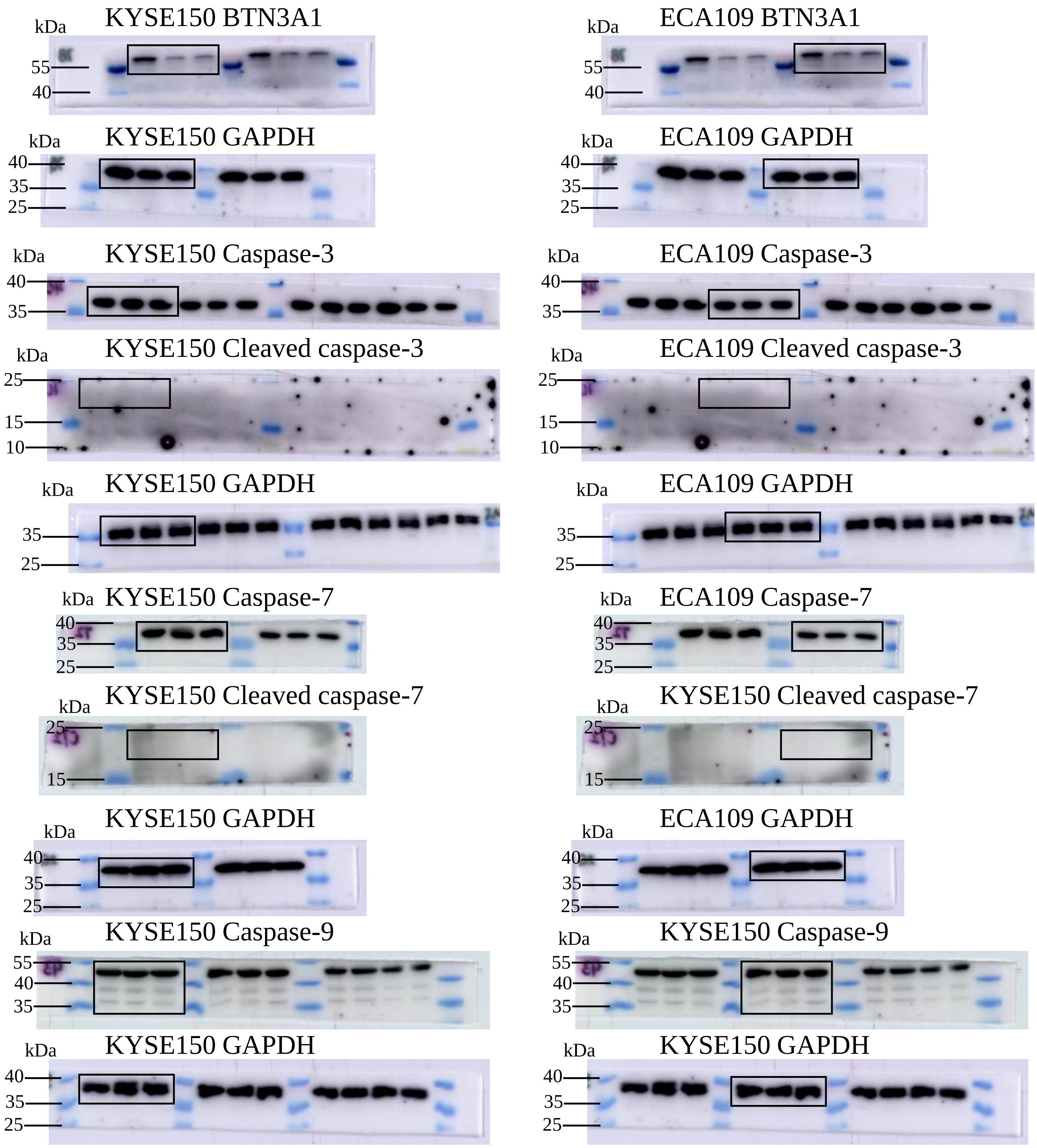

Fig.S5C

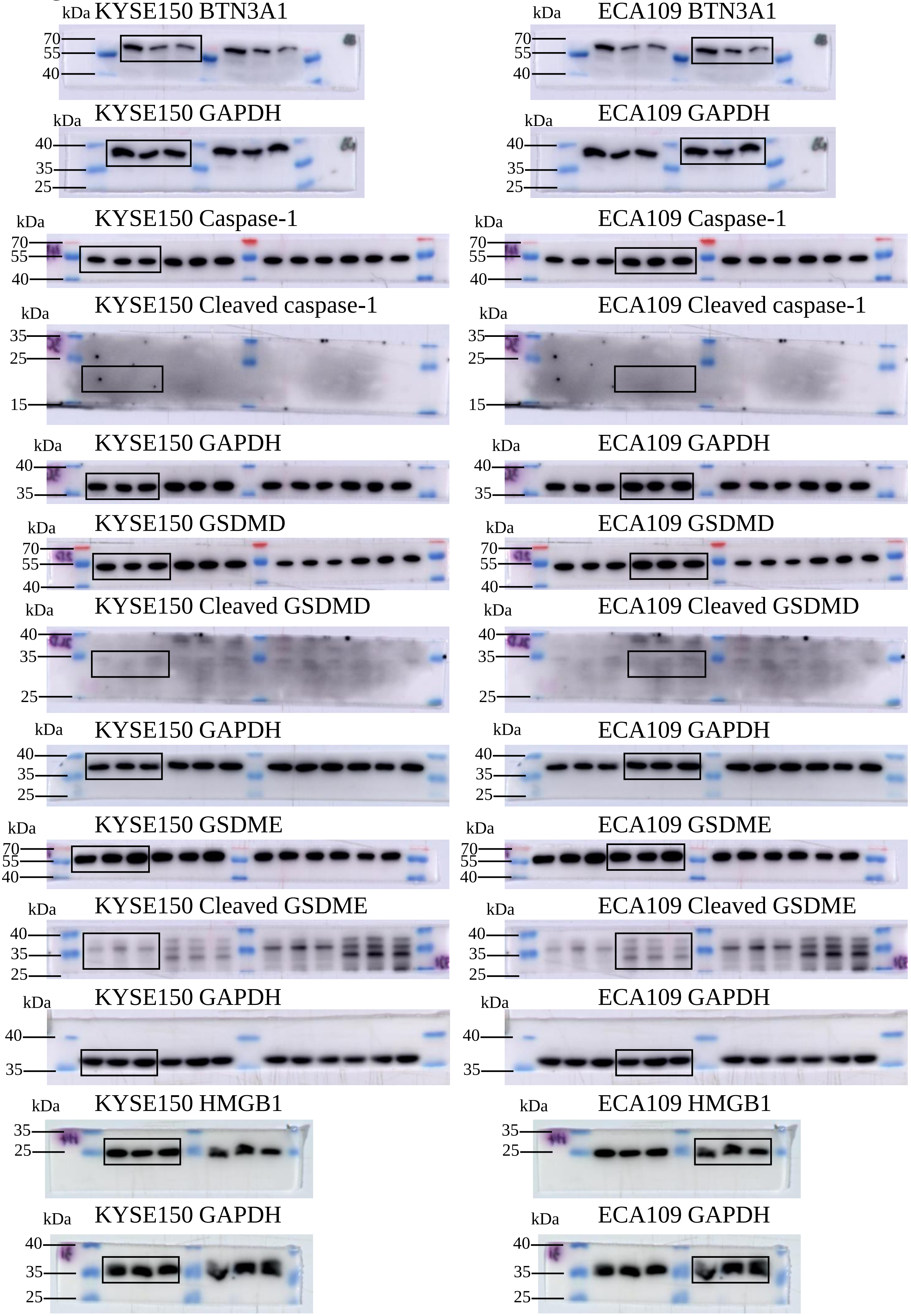
KYSE150 BTN3A1
ECA109 BTN3A1
KYSE150 GAPDH
ECA109 GAPDH
KYSE150 Caspase-1
ECA109 Caspase-1
KYSE150 Cleaved caspase-1
ECA109 Cleaved caspase-1
KYSE150 GAPDH
ECA109 GAPDH
KYSE150 GSDMD
ECA109 GSDMD
KYSE150 Cleaved GSDMD
ECA109 Cleaved GSDMD
KYSE150 GAPDH
ECA109 GAPDH
KYSE150 GSDME
ECA109 GSDME
KYSE150 Cleaved GSDME
ECA109 Cleaved GSDME
KYSE150 GAPDH
ECA109 GAPDH
KYSE150 HMGB1
ECA109 HMGB1
KYSE150 GAPDH
ECA109 GAPDH
kDa
70
55
40
35
25
15

Uncropped blots related to Figure.S5&S6

Fig.S5D

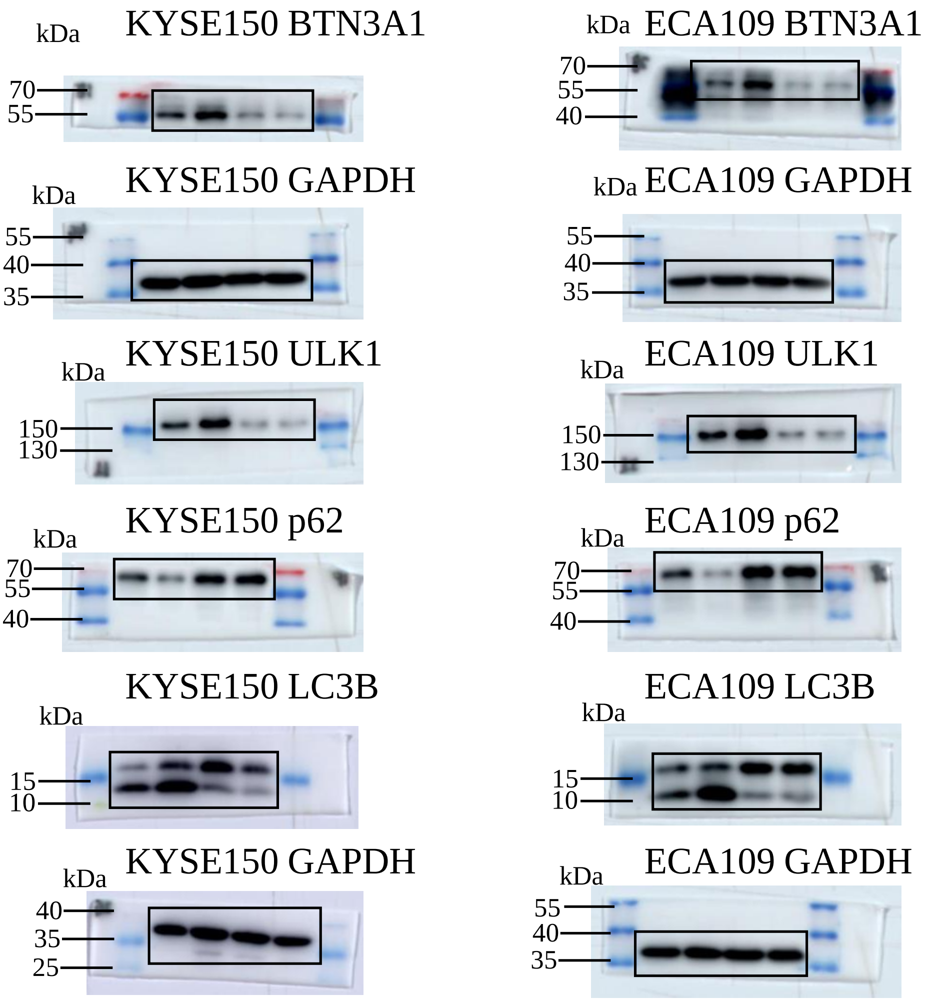

Fig.S5G

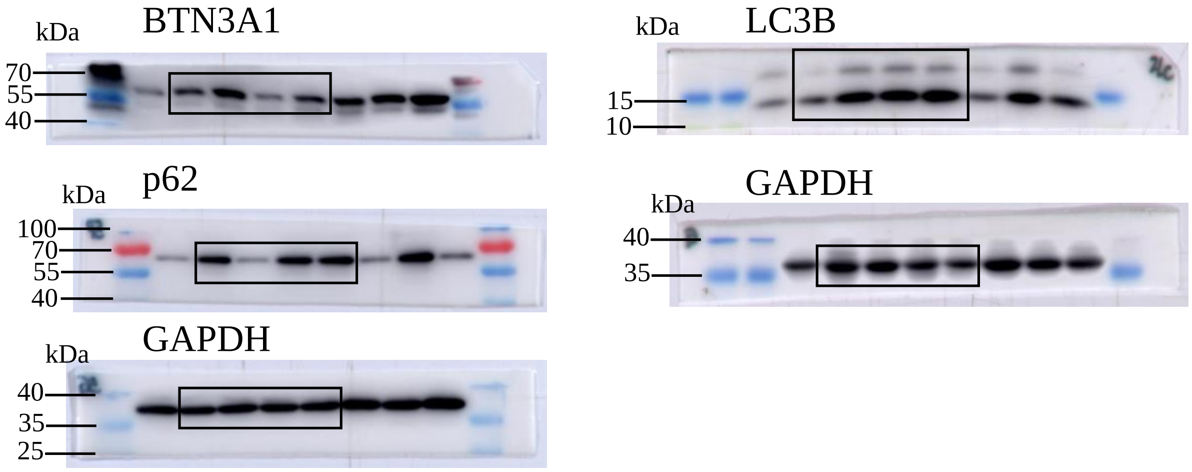

Fig.S6B

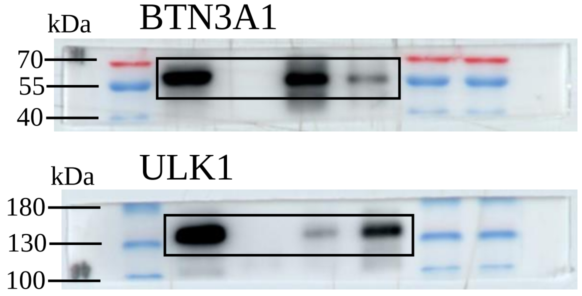

Uncropped blots related to Figure.S6

Fig.S6E

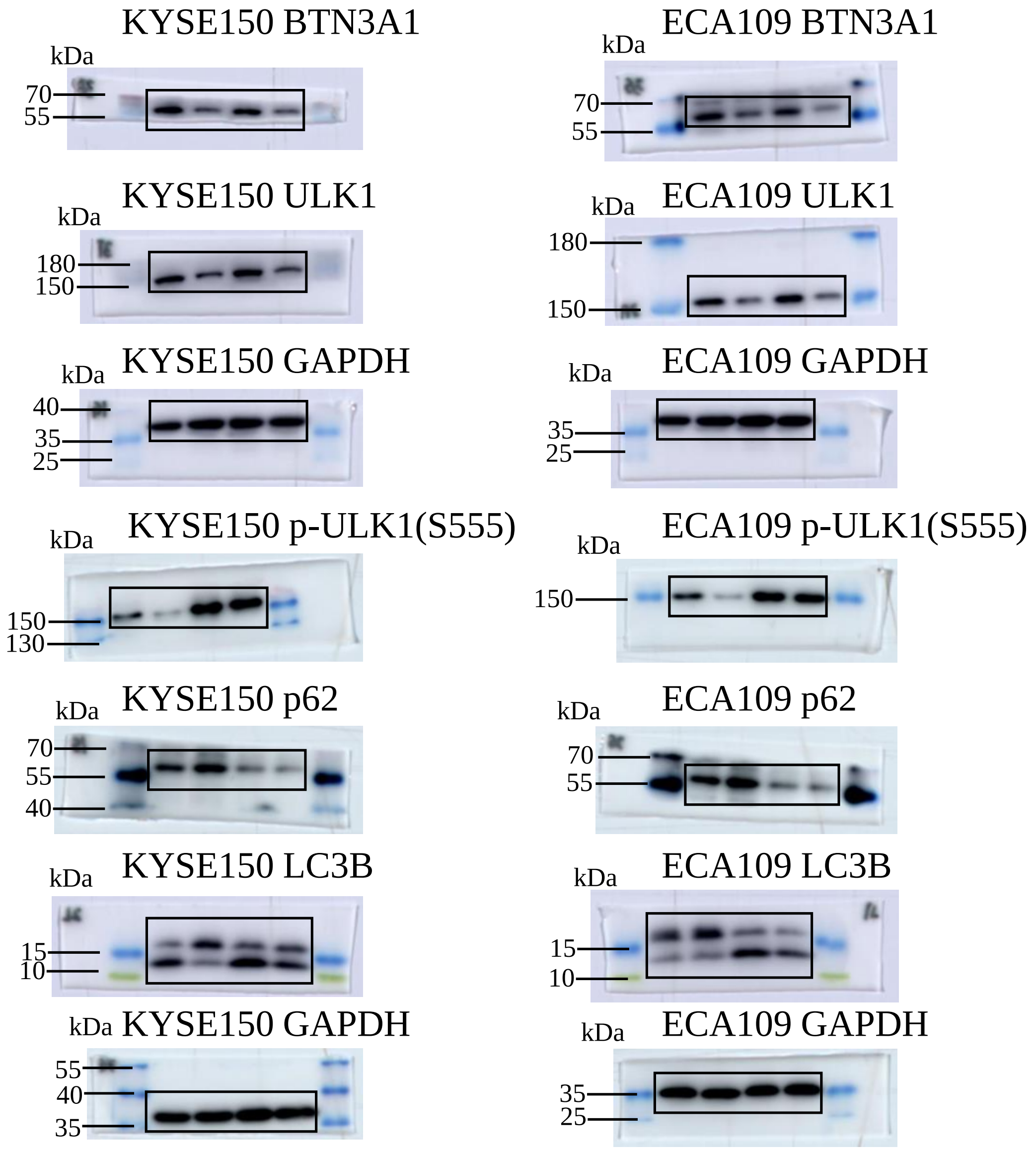

Fig.S6H

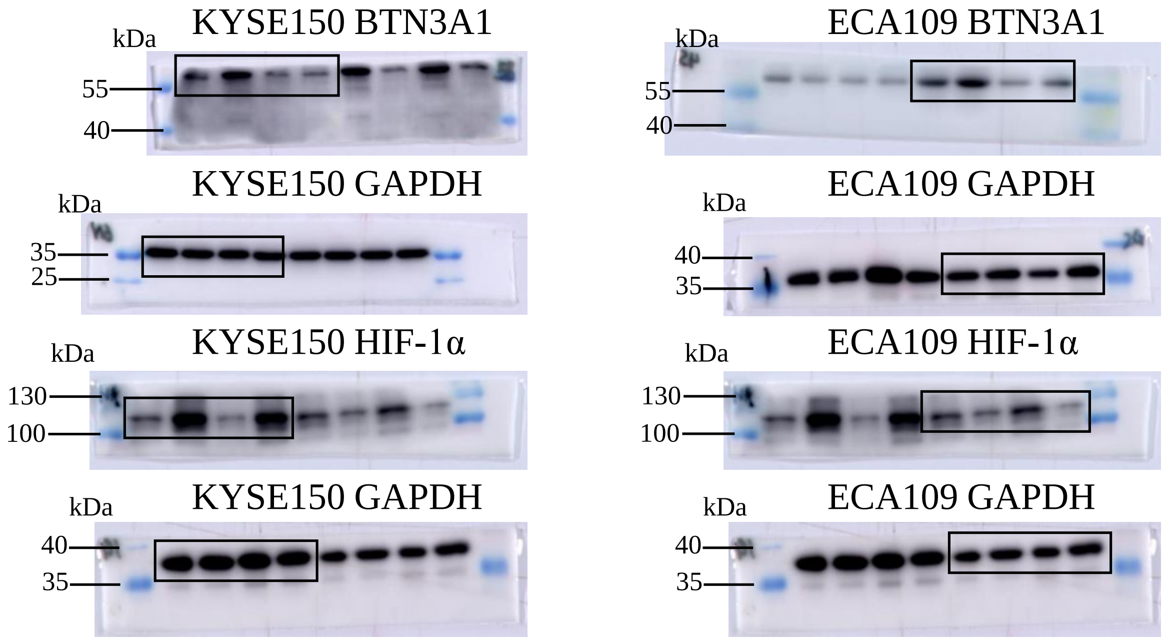

Supplement: Supplementary file 19 — Uncropped Western blots [file 41419_2022_5429_MOESM19_ESM.pdf]
