# Supplementary material for: BTN3A1 promotes tumor progression and radiation resistance in esophageal squamous cell carcinoma by regulating ULK1-mediated autophagy
Source: Cell Death Dis. 2022 Nov 22;13(11):984. doi: 10.1038/s41419-022-05429-w (PMC9684582; doi:10.1038/s41419-022-05429-w)
Supplement: Supplementary file 20 — Author Contribution Statement [file 41419_2022_5429_MOESM20_ESM.docx]

**Author Contribution Statement**

Y.-F.C. performed the conception and design of this manuscript. B.C. and P.-X.C. collected clinical tumor samples and vitro experiments. W.-J.Y. and Z.-H.W. performed data analysis and vitro experiments. W.-J.Y. performed vivo experiments. W.-J.Y. and X.-Z.S performed the manuscript writing. Z.-H.W. and P.-X.C.revised the manuscript. All authors were involved in writing the paper and had final approval of the final manuscript.
