# Supplementary material for: BTN3A1 promotes tumor progression and radiation resistance in esophageal squamous cell carcinoma by regulating ULK1-mediated autophagy
Source: Cell Death Dis. 2022 Nov 22;13(11):984. doi: 10.1038/s41419-022-05429-w (PMC9684582; doi:10.1038/s41419-022-05429-w)
Supplement: Supplementary file 21 — Related Manuscript Information: Vector backbone information [file 41419_2022_5429_MOESM21_ESM.docx]

| Vector backbone information |
| --- |
| 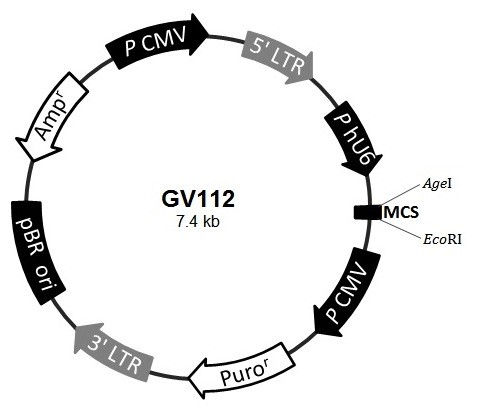 |
| Vector information for GV112 |
| 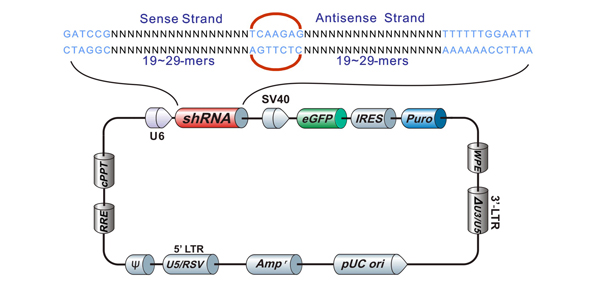 |
| Vector information for CS-SH3295-LVRU6GP |
| 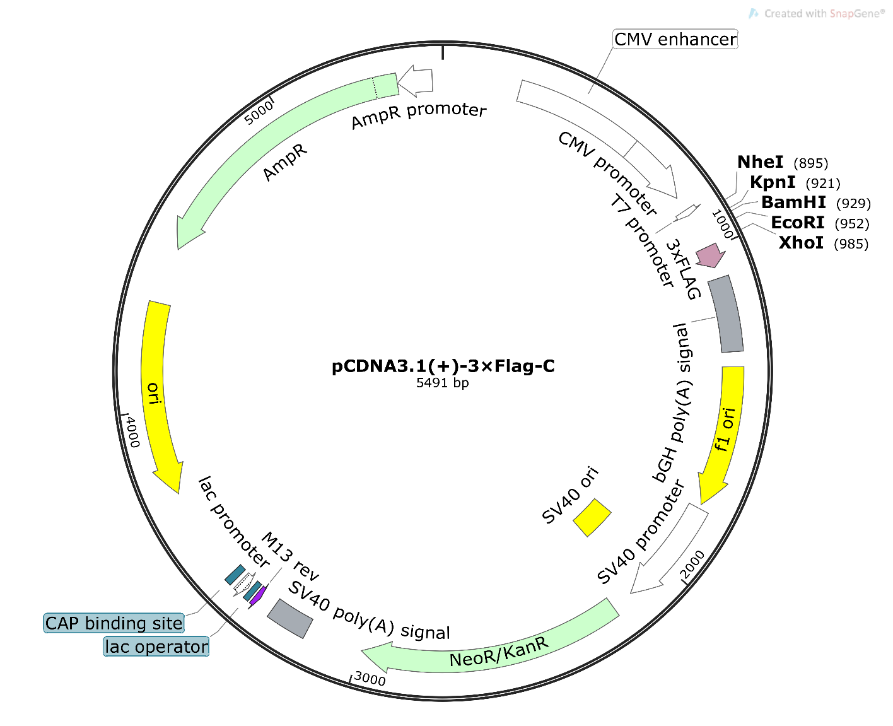 |
| Vector information for Pcdna3.1(+)-3×Flag-C |
| 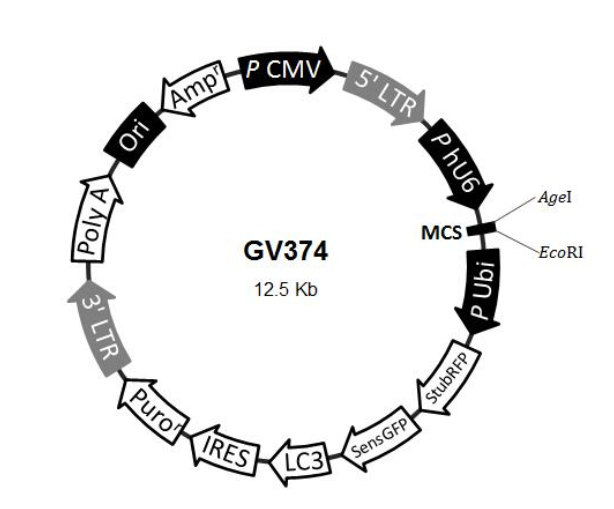 |
| Vector information for mRFP-GFP-LC3 |
| 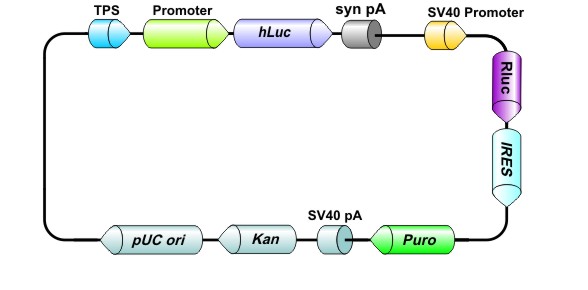 |
| Vector Information for PL01 |
| 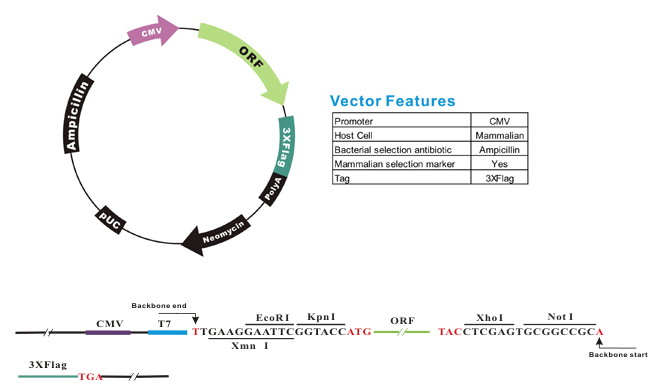 |
| Vector Information for PM14 |
