# Supplementary material for: BTN3A1 promotes tumor progression and radiation resistance in esophageal squamous cell carcinoma by regulating ULK1-mediated autophagy
Source: Cell Death Dis. 2022 Nov 22;13(11):984. doi: 10.1038/s41419-022-05429-w (PMC9684582; doi:10.1038/s41419-022-05429-w)
Supplement: Supplementary file 22 — Cell Line Authentication - STR Profiling Report [file 41419_2022_5429_MOESM22_ESM.pdf]

Eca-109 细胞 STR 鉴定报告

一、 材料处理和检验方法

取适量 **Eca-109** 细胞(1×10^6)使用 PureLink® Genomic DNA Mini Kit（美国 Life K182001）提前基因组 DNA，采用 PowerPlex®18D 系统（美国 Promega DC1802）试剂盒进行扩增，在 ABI3500 Genetic Analyzer（美国 Life3500）进行检测。

二、 检测结果

实验中阴性及阳性对照结果均正确。

Eca-109 细胞株的 STR 位点和 Amelogenin 位点的基因分型结果见附表，分型图谱见附图。

三、 分析说明

Eca-109 细胞株基因组 DNA 扩增后图谱清晰，分型结果良好。

四、 检验结论

1. **Eca-109** 细胞株 DNA 进行细胞 STR 分型结果显示，细胞株中未发现人类细胞交叉污染。
2. 该细胞株 DNA 分型在 CRC 细胞库中找到与其细胞分型 100%相匹配的细胞株，细胞株名称为 **Eca-109**。

附图 1：Eca-109 细胞 STR 位点和 Amelogenin 位点的基因分型结果

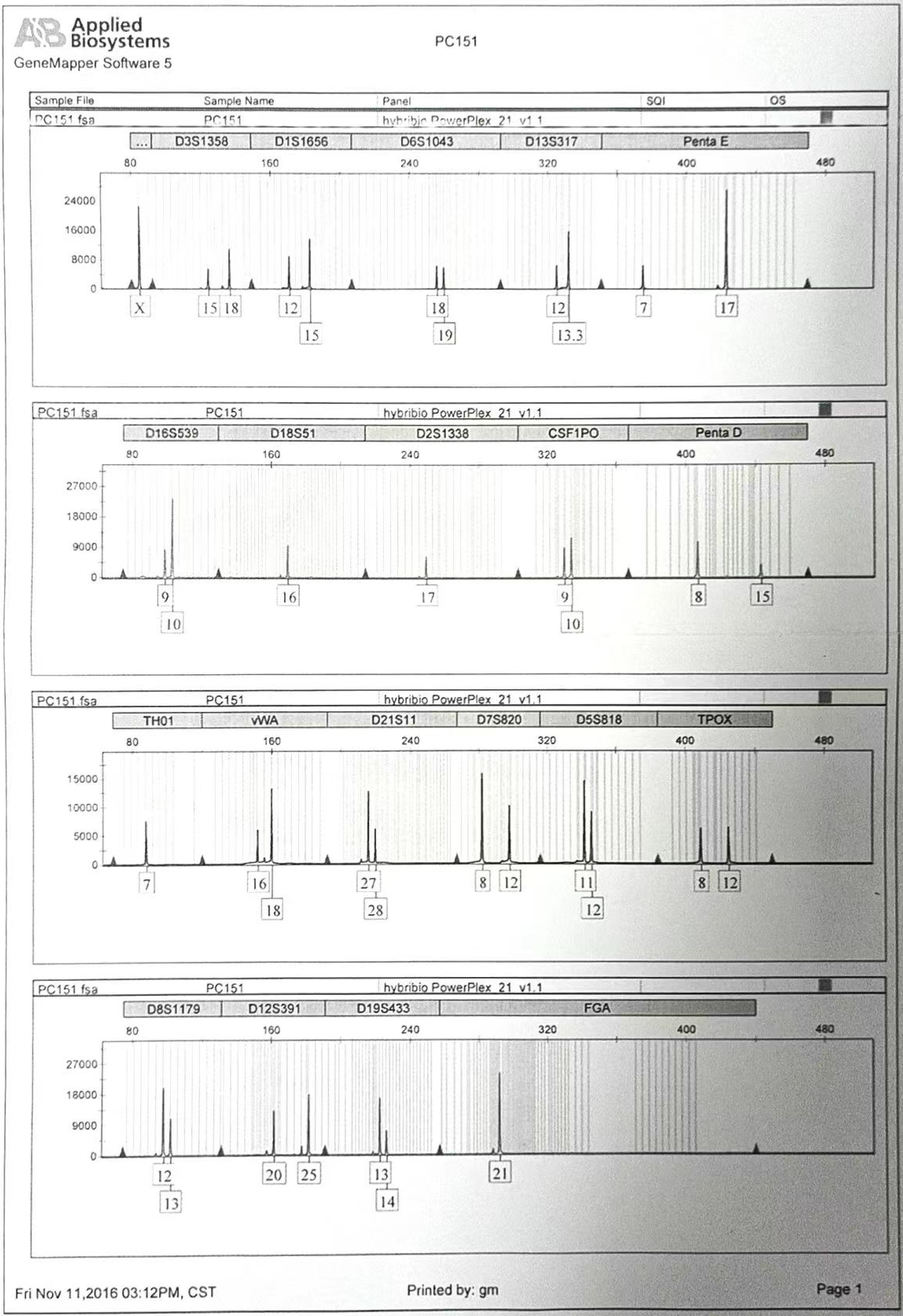

网站：www.procell.com.cn
电话：400-650-3656
邮箱：sales@procell.com.cn

武汉普诺赛生命科技有限公司

Procell Life Science&Technology Co,.Ltd.

附表 1：细胞株 Eca-109 的 STR 位点和 Amelogenin 位点的基因分型结果

细胞 **Eca-109**（图片编号为 PC151）		
Marker	***Allele 1***	***Allele 2***
D3S1358	15	18
**THO1**	7	
D21S11	27	28
D18S51	16	
Penta E	7	17
**D5S818**	11	12
**D13S317**	12	13.3
**D7S820**	8	12
**D16S539**	9	10
**CSF1PO**	9	10
Penta D	8	15
**AMEL**	X	
**Vwa**	16	18
D8S1179	12	13
**TPOX**	8	12
FGA	21	
D19S433	13	14
D2S1338	17	
D1S1656	12	15
D6S1043	18	19
D12S391	20	25

Cell Line Authentication – STR Profiling Report

Sample Type: Cell Line
Testing Type: STR

Sample code:

Table 1. Sample Code

Customer’s code	Company Code
5	20201019-02

Sample Number:1

Sample Type: Cell line

Testing Type: STR

Sample From: Shanghai Zhong Qiao Xin Zhou Biotechnology Co.,Ltd.

Testing Method:

DNA was extracted by a commercial kit from CORNING (AP-EMN-BL-GDNA-250G). Twenty short tandem repeat (STR) loci plus the gender determining locus, Amelogenin, were amplified by six multiplex PCR and separated on ABI 3730XL Genetic Analyzer. The signals were then analyzed by the software GeneMapper.

Data Interpretation:

Cell lines were authenticated using Short Tandem Repeat (STR) analysis as described in 2012 in ANSI Standard (ASN-0002) by the ATCC Standards Development Organization (SDO) and in Capes-Davis et al., Match criteria for human cell line authentication: Where do we draw the line? Int J Cancer. 2013;132(11):2510-9.

Test Results:

1. Result

Table 2. Matching information on the cell lines

Sample Code	Multi-allele	Cell line matched	Cell Bank	EV
20201019-02	NO	KYSE-150	DSMZ	1.0

- Multi-allele means some STR contain more than two loci.

2. Sample Description

20201019-02:

A. The STR results showed that no multiple alleles were found in this cell line, and no cross contamination of human cells was found in the cell line.

B. The DNA of the cell lines found to match the type of cell lines in a cell line retrieval, DSMZ database shows that cells called **KYSE-150,** corresponding to the cell number **375.**

3. Genotyping Result

Table 3. STR and Amelogenin Genotyping Results of Cell line 20201019-02

Loci	Sample information			Cell Bank information		
	Sample name：5			Cell line name：KYSE-150		
	**Allele1**	**Allele2**	**Allele3**	**Allele1**	**Allele2**	**Allele3**
D5S818	12	13		12	13	
D13S317	8	11		8	11	
D7S820	10	11		10	11	
D16S539	9	11		9	11	

VWA	16	17	16	17
TH01	7	9	7	9
AMEL	X	X	X	X
TPOX	8	8	8	8
CSF1PO	12	13	12	13
D12S391	19	22		
FGA	21	24		
D2S1338	25	25		
D21S11	30	31		
D18S51	14	14		
D8S1179	10	15		
D3S1358	15	16		
D6S1043	18	20		
PENTAE	12	18		
D19S433	15	15.2		
PENTAD	10	10		
D1S1656	15	17.3		

The allele match algorithm compares the 8 core loci plus amelogenin only, even though alleles from all loci will be reported when available.

Others:

1. Genotyping Strategy and Site Distribution

Attached Table. Experimental Strategy and Sites

	Strategy 1	Strategy 2	Strategy 3	Strategy 4
1	D3S1358	D8S1179	D19S433	AMEL
2	VWA	D21S11	TH01	D1S1656
3	D7S820	D16S539	D13S317	D5S818
4	CSF1PO	D2S1338	TPOX	D12S391
5	PENTAE	PENTAD	D18S51	FGA
6			D6S1043	

2. DSMZ tools was used to carry on the cell line comparison, which contains 2455 cell lines STR data from ATCC, DSMZ, JCRB, ECACC, GNE and RIKEN databases. If the cell is not included in the above cell library, users need to compared with other databases.

Certificate of STR Analysis

Applied Biosystems
GeneMapper ID v3.2

20201019

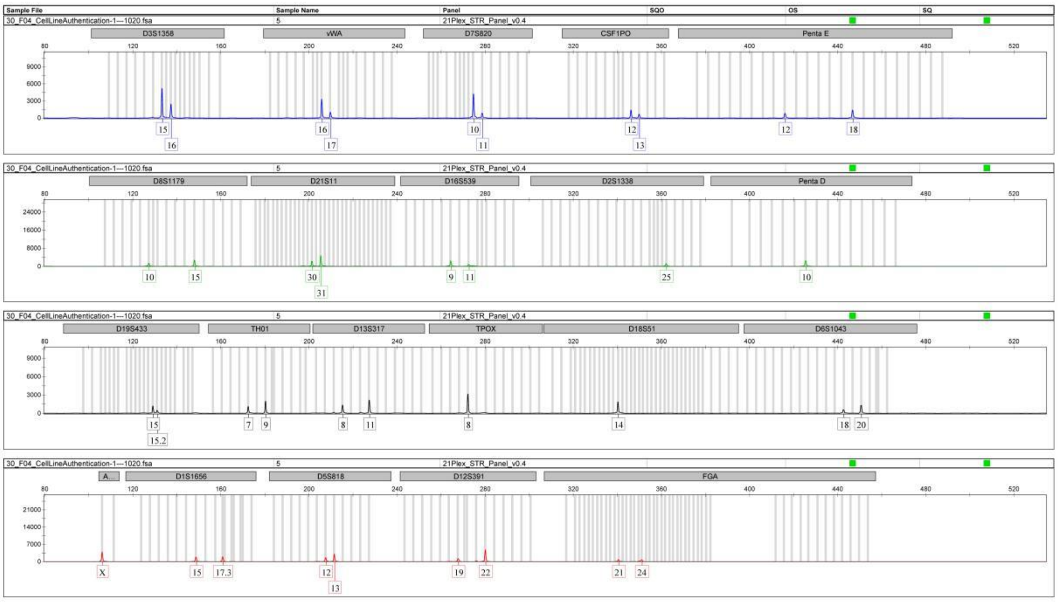

Wed Oct 21,2020 05:47PM, GMT+08:00

Printed by: gmid

Report Date:
Oct., 2020

Cell Line Authentication – STR Profiling Report

Sample Type: Cell Line
Testing Type: STR

Sample code:

Table 1. Sample Code

Customer's code	Company Code
A	20201102-01

Sample Number:1

Sample Type: Cell line

Testing Type: STR

Sample From: Shanghai Zhong Qiao Xin Zhou Biotechnology Co.,Ltd.

Testing Method:

DNA was extracted by a commercial kit from CORNING (AP-EMN-BL-GDNA-250G). Twenty short tandem repeat (STR) loci plus the gender determining locus, Amelogenin, were amplified by six multiplex PCR and separated on ABI 3730XL Genetic Analyzer. The signals were then analyzed by the software GeneMapper.

Data Interpretation:

Cell lines were authenticated using Short Tandem Repeat (STR) analysis as described in 2012 in ANSI Standard (ASN-0002) by the ATCC Standards Development Organization (SDO) and in Capes-Davis et al., Match criteria for human cell line authentication: Where do we draw the line? Int J Cancer. 2013;132(11):2510-9.

Test Results:

1. Result

Table 2. Matching information on the cell lines

Sample Code	Multi-allele	Cell line matched	Cell Bank	EV
20201102-01	No	KYSE-510	DSMZ	1.0

- Multi-allele means some STR contain more than two loci.

2. Sample Description

20201102-01:

A. The STR results showed that no multiple alleles were found in this cell line, and no cross contamination of human cells was found in the cell line.

B. The DNA of the cell lines found to match the type of cell lines in a cell line retrieval, DSMZ database shows that cells called **KYSE-510,** corresponding to the cell number **374.**

3. Genotyping Result

Table 3. STR and Amelogenin Genotyping Results of Cell line 20201102-01

Loci	Sample information			Cell Bank information		
	Sample name：A			Cell line name：KYSE-510		
	**Allele1**	**Allele2**	**Allele3**	**Allele1**	**Allele2**	**Allele3**
D5S818	11	11		11	11	
D13S317	12	12		12	12	
D7S820	11	12		11	12	
D16S539	9	9		9	9	

VWA	14	14	14	14
TH01	9	9	9	9
AMEL	X	X	X	X
TPOX	8	8	8	8
CSF1PO	11	13	11	13
D12S391	18	21		
FGA	22	22		
D2S1338	20	23		
D21S11	31	31		
D18S51	15	15		
D8S1179	10	10		
D3S1358	17	17		
D6S1043	14	14		
PENTAE	16	16		
D19S433	15	15		
PENTAD	12	12		
D1S1656	13	13		

The allele match algorithm compares the 8 core loci plus amelogenin only, even though alleles from all loci will be reported when available.

Others:

1. Genotyping Strategy and Site Distribution

Attached Table. Experimental Strategy and Sites

	Strategy 1	Strategy 2	Strategy 3	Strategy 4
1	D3S1358	D8S1179	D19S433	AMEL
2	VWA	D21S11	TH01	D1S1656
3	D7S820	D16S539	D13S317	D5S818
4	CSF1PO	D2S1338	TPOX	D12S391
5	PENTAE	PENTAD	D18S51	FGA
6			D6S1043	

2. DSMZ tools was used to carry on the cell line comparison, which contains 2455 cell lines STR data from ATCC, DSMZ, JCRB, ECACC, GNE and RIKEN databases. If the cell is not included in the above cell library, users need to compared with other databases.

中乔新舟
CELL RESEARCH

Certificate of STR Analysis

Applied Biosystems
GeneMapper ID v3.2

20201102

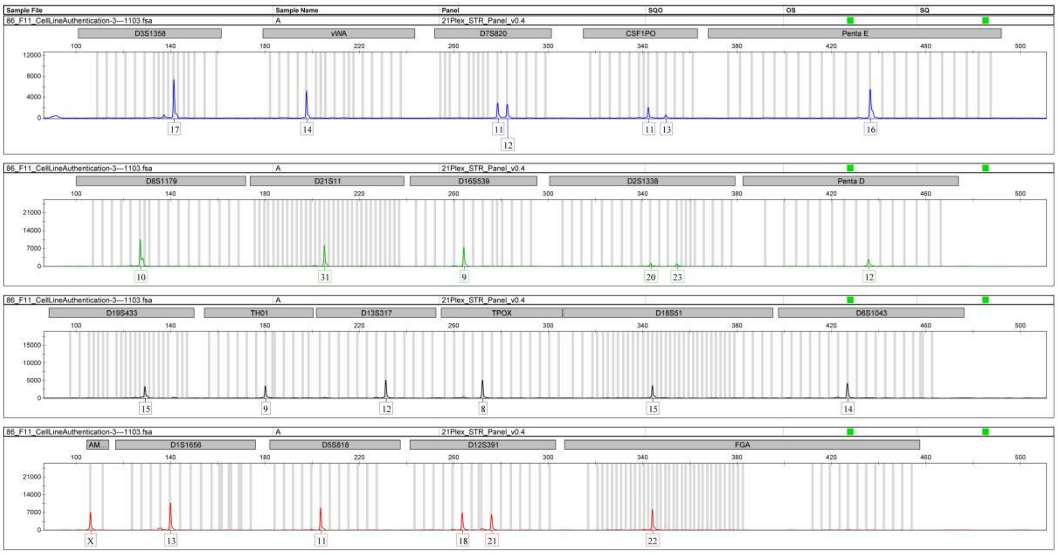

Wed Nov 04,2020 03:57PM, GMT+08:00

Printed by: gmid

Page 1 of 1

Report Date:
Nov., 2020

Cell Line Authentication – STR Profiling Report

Sample Type: Cell Line
Testing Type: STR

Sample code:

Table 1. Sample Code

Customer's code	Company Code
4	20201019-01

Sample Number:1

Sample Type: Cell line

Testing Type: STR

Sample From: Shanghai Zhong Qiao Xin Zhou Biotechnology Co.,Ltd.

Testing Method:

DNA was extracted by a commercial kit from CORNING (AP-EMN-BL-GDNA-250G). Twenty short tandem repeat (STR) loci plus the gender determining locus, Amelogenin, were amplified by six multiplex PCR and separated on ABI 3730XL Genetic Analyzer. The signals were then analyzed by the software GeneMapper.

Data Interpretation:

Cell lines were authenticated using Short Tandem Repeat (STR) analysis as described in 2012 in ANSI Standard (ASN-0002) by the ATCC Standards Development Organization (SDO) and in Capes-Davis et al., Match criteria for human cell line authentication: Where do we draw the line? Int J Cancer. 2013;132(11):2510-9.

Test Results:

1. Result

Table 2. Matching information on the cell lines

Sample Code	Multi-allele	Cell line matched	Cell Bank	EV
20201019-01	NO	KYSE-140	DSMZ	1.0

- Multi-allele means some STR contain more than two loci.

2. Sample Description

20201019-01:

A. The STR results showed that no multiple alleles were found in this cell line, and no cross contamination of human cells was found in the cell line.

B. The DNA of the cell lines found to match the type of cell lines in a cell line retrieval, DSMZ database shows that cells called **KYSE-140,** corresponding to the cell number **348.**

3. Genotyping Result

Table 3. STR and Amelogenin Genotyping Results of Cell line 20201019-01

Loci	Sample information			Cell Bank information		
	Sample name：4			Cell line name：KYSE-140		
	**Allele1**	**Allele2**	**Allele3**	**Allele1**	**Allele2**	**Allele3**
D5S818	10	10		10	10	
D13S317	12	12		12	12	
D7S820	10	10		10	10	
D16S539	10	12		10	12	

VWA	14	14	14	14
TH01	7	9	7	9
AMEL	X	X	X	X
TPOX	8	8	8	8
CSF1PO	13	13	13	13
D12S391	17	19		
FGA	22	23		
D2S1338	17	17		
D21S11	29	29		
D18S51	14	14		
D8S1179	10	10		
D3S1358	18	18		
D6S1043	11	14		
PENTAE	16	17		
D19S433	13	15		
PENTAD	9	9		
D1S1656	17	17		

The allele match algorithm compares the 8 core loci plus amelogenin only, even though alleles from all loci will be reported when available.

Others:

1. Genotyping Strategy and Site Distribution

Attached Table. Experimental Strategy and Sites

	Strategy 1	Strategy 2	Strategy 3	Strategy 4
1	D3S1358	D8S1179	D19S433	AMEL
2	VWA	D21S11	TH01	D1S1656
3	D7S820	D16S539	D13S317	D5S818
4	CSF1PO	D2S1338	TPOX	D12S391
5	PENTAE	PENTAD	D18S51	FGA
6			D6S1043	

2. DSMZ tools was used to carry on the cell line comparison, which contains 2455 cell lines STR data from ATCC, DSMZ, JCRB, ECACC, GNE and RIKEN databases. If the cell is not included in the above cell library, users need to compared with other databases.

中乔新舟
CELL RESEARCH

Certificate of STR Analysis

Applied Biosystems
GeneMapper ID v3.2

20201019

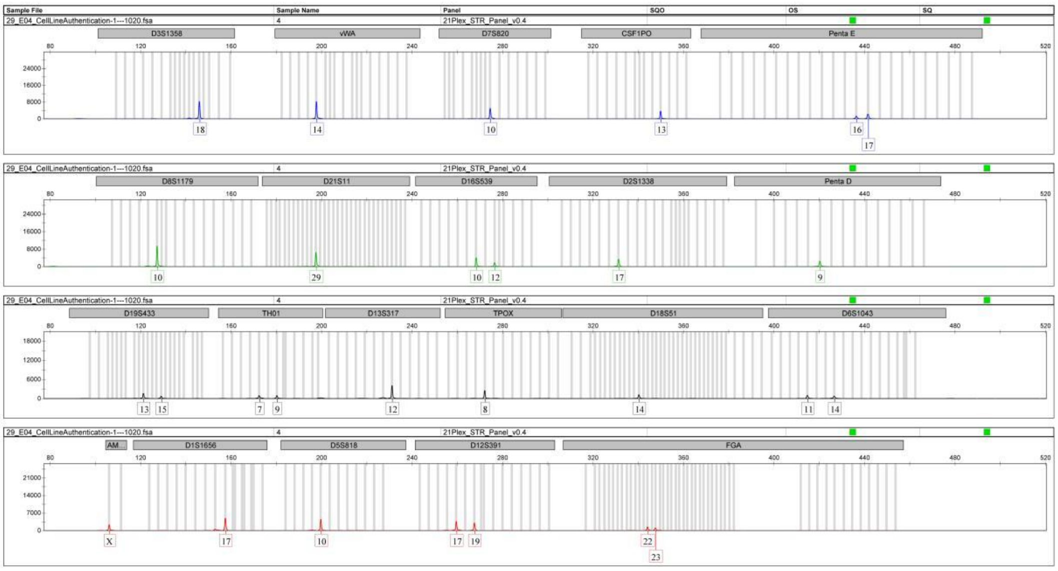

Report Date:
Oct., 2020

Cell Line Authentication – STR Profiling Report

Sample Type: Cell Line
Testing Type: STR

Sample code:

Table 1. Sample Code

Customer's code	Company Code
876	20210607-01

Sample Number:1

Sample Type: Cell line

Testing Type: STR

Sample From: Shanghai Zhong Qiao Xin Zhou Biotechnology Co.,Ltd.

Testing Method:

DNA was extracted by a commercial kit from CORNING (AP-EMN-BL-GDNA-250G). Twenty short tandem repeat (STR) loci plus the gender determining locus, Amelogenin, were amplified by six multiplex PCR and separated on ABI 3730XL Genetic Analyzer. The signals were then analyzed by the software GeneMapper.

Data Interpretation:

Cell lines were authenticated using Short Tandem Repeat (STR) analysis as described in 2012 in ANSI Standard (ASN-0002) by the ATCC Standards Development Organization (SDO) and in Capes-Davis et al., Match criteria for human cell line authentication: Where do we draw the line? Int J Cancer. 2013;132(11):2510-9.

Test Results:

1. Result

Table 2. Matching information on the cell lines

Sample Code	Multi-allele	Cell line matched	Cell Bank	EV
20210607-01	NO	TE-1	DSMZ	1.0

- Multi-allele means some STR contain more than two loci.

2. Sample Description

20210607-01:

A. The STR results showed that no multiple alleles were found in this cell line, and no cross contamination of human cells was found in the cell line.

B. The DNA of the cell lines found to match the type of cell lines in a cell line retrieval, DSMZ database shows that cells called **TE-1,** corresponding to the cell number **RCB1894.**

3. Genotyping Result

Table 3. STR and Amelogenin Genotyping Results of Cell line 20210607-01

Loci	Sample information			Cell Bank information		
	Sample name：876			Cell line name：TE-1		
	**Allele1**	**Allele2**	**Allele3**	**Allele1**	**Allele2**	**Allele3**
D5S818	11	11		11	11	
D13S317	10	10		10	10	

D7S820	10	11	10	11
D16S539	12	12	12	12
VWA	17	18	17	18
TH01	7	7	7	7
AMEL	X	X	X	X
TPOX	8	11	8	11
CSF1PO	10	12	10	12
D12S391	20	24		
FGA	24	24		
D2S1338	19	20		
D21S11	28	28		
D18S51	17	17		
D8S1179	11	13		
D3S1358	16	16		
D6S1043	11	12		
PENTAE	12	18		
D19S433	14	15.2		
PENTAD	10	10		
D1S1656	11	15		

The allele match algorithm compares the 8 core loci plus amelogenin only, even though alleles from all loci will be reported when available.

Others:

1. Genotyping Strategy and Site Distribution

Attached Table. Experimental Strategy and Sites

	Strategy 1	Strategy 2	Strategy 3	Strategy 4
1	D3S1358	D8S1179	D19S433	AMEL
2	VWA	D21S11	TH01	D1S1656
3	D7S820	D16S539	D13S317	D5S818
4	CSF1PO	D2S1338	TPOX	D12S391
5	PENTAE	PENTAD	D18S51	FGA
6			D6S1043	

2. DSMZ tools was used to carry on the cell line comparison, which contains 2455 cell lines STR data from ATCC, DSMZ, JCRB, ECACC, GNE and RIKEN databases. If the cell is not included in the above cell library, users need to compared with other databases.

Certificate of STR Analysis

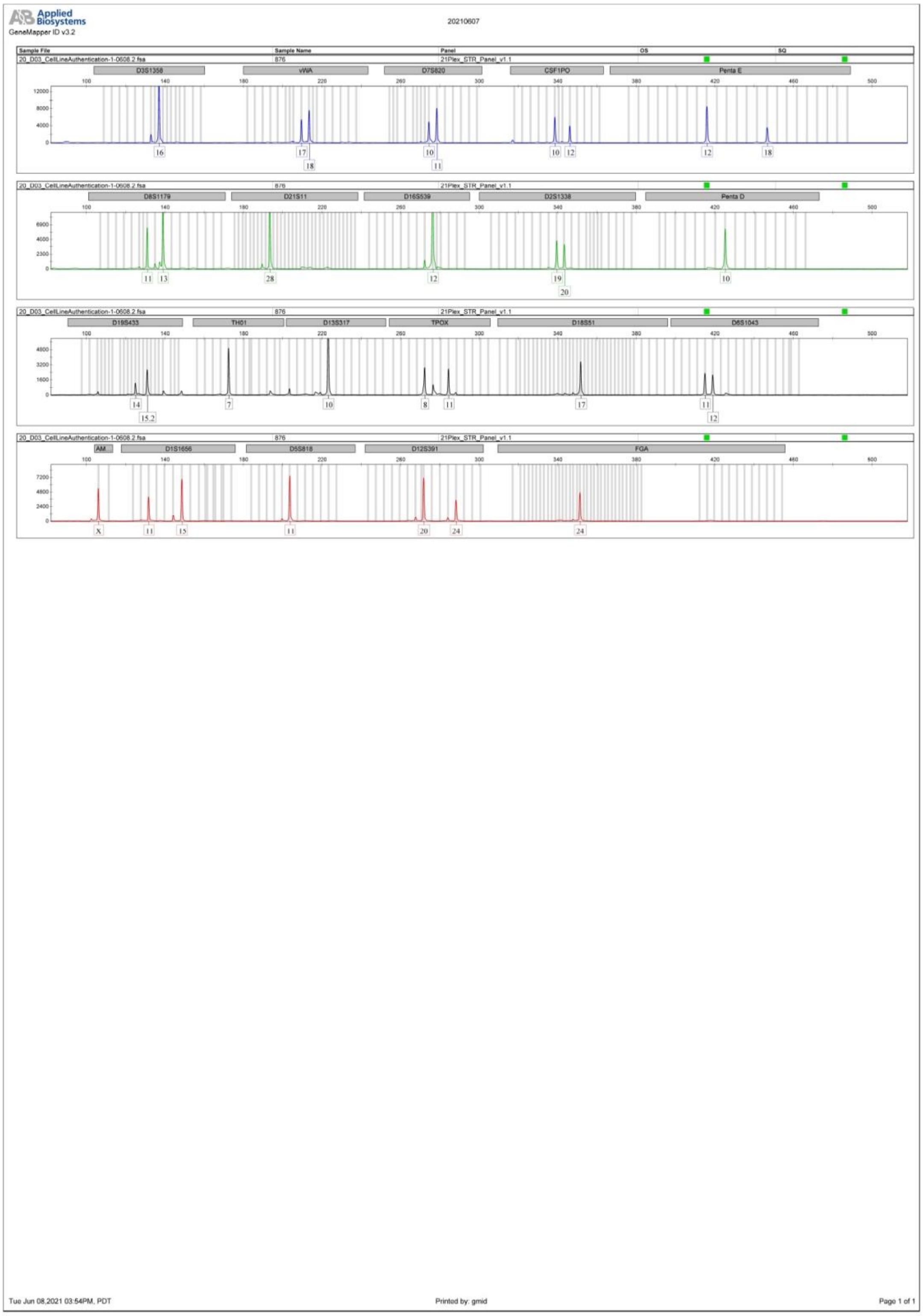

Report Date:
Jun., 2021
